# Functionalization Techniques Empowering Optical Fiber Biosensors in Label-Free Cancer Biomarker Detection

**DOI:** 10.3390/bios16010025

**Published:** 2025-12-31

**Authors:** Aigerim Omirzakova, Lyazzat Mukhangaliyeva, Zhanerke Katrenova, Aida Aituganova, Aliya Bekmurzayeva, Daniele Tosi, Zhannat Ashikbayeva

**Affiliations:** 1 Laboratory of Biosensors and Bioinstruments, National Laboratory Astana, Astana 010000, Kazakhstan; aigerim.omirzakova@nu.edu.kz (A.O.); lyazzat.mukhangaliyeva@nu.edu.kz (L.M.); zh.katrenova@astanait.edu.kz (Z.K.); abekmurzayeva@nu.edu.kz (A.B.); daniele.tosi@nu.edu.kz (D.T.); 2 Department of Chemical and Materials Science Engineering, School of Engineering and Digital Sciences, Nazarbayev University, Astana 010000, Kazakhstan; aida.aituganova@nu.edu.kz; 3 Department of Science and Innovation, Astana IT University, Astana 010000, Kazakhstan; 4 Department of Electrical and Computer Engineering, School of Engineering and Digital Sciences, Nazarbayev University, Astana 010000, Kazakhstan; 5 Department of Chemistry, School of Sciences and Humanities, Nazarbayev University, Astana 010000, Kazakhstan

**Keywords:** optical fiber biosensors, cancer detection, biomedical diagnostic technologies, label-free detection, surface modification, silanization, nanomaterials, cancer biomarkers

## Abstract

Optical fibers are gaining increasing attention in biomedical applications due to their unique advantages, including flexibility, biocompatibility, immunity to electromagnetic interference, potential for miniaturization, and the ability to perform remote, real-time, and in situ sensing. Label-free optical fiber biosensors represent a promising alternative to conventional cancer diagnostics, offering comparable sensitivity and specificity while enabling real-time detection at ultra-low concentrations without the need for complex labeling procedures. However, the sensing performance of biosensors is fundamentally governed by surface modification. The choice of optimal functionalization strategy is dictated by the sensor type, target biomarker, and detection environment. This review paper presents a comprehensive and expanded overview of various surface functionalization methods specifically designed for cancer biomarker detection using optical fiber biosensors, including silanization, self-assembled monolayers, polymer-based coatings, and different dimensional nanomaterials (0D, 1D, and 2D). Furthermore, the emerging integration of computational methods and machine learning in optimizing functionalized optical sensing has been discussed. To the best of our knowledge, this is the first work that consolidates existing surface modification approaches into a single, cohesive resource, providing valuable insights for researchers developing next-generation fiber optic biosensors for cancer diagnostics. Moreover, the paper points out the current technical challenges and outlines the future perspectives of optical fiber-based biosensors.

## 1. Introduction

Cancer is a non-communicable disease that does not discriminate based on gender, age, social status, or economic background. It manifests as the uncontrollable growth of abnormal cells and their ability to spread rapidly to other parts of the body. According to World Health Organization’s (WHO) statistics, cancer was responsible for approximately 10 million deaths worldwide in 2020 [1]. Approximately 50% of cancer cases are diagnosed at advanced stages, when patients have limited treatment options [2]. Therefore, diagnosing cancer at an early stage, given that an effective treatment is provided in a timely manner, can substantially increase the chances of survival. Traditional methods to diagnose cancer include but are not limited to tissue biopsy, magnetic resonance imaging (MRI), ultrasound imaging, enzyme-linked immunosorbent assays (ELISA), and others. However, these techniques often have significant limitations, such as high cost of equipment and consumables, the need for highly trained operators, limited resolution, large sample volume requirements, and invasive nature of some procedures, which can cause patient discomfort [3]. Additionally, these techniques are often unsuitable for detecting cancer at an early stage, as the size of the tumor is significantly small and patients typically show no symptoms. Therefore, it is crucial to develop early detection methods for diagnosing cancer that are reliable, cost-effective, and preferably minimally or completely non-invasive, ensuring accessibility for all populations and maximizing patient comfort.

Currently, various early cancer detection approaches are being explored [4], and among them, optical fiber-based biosensors (OFBs) show promising results by offering high sensitivity, affordability, biocompatibility, and user-friendly operation [5]. Core, cladding, and a coating are the main parts of an optical fiber. Silica and plastic optical fibers (POFs) operate on the principle of total internal reflection, achieved by a core–cladding structure with a defined refractive index (RI) difference. In silica fibers, both components are typically made of silicon dioxide with dopants used to ensure a higher RI in the core [6]. POFs utilize high-purity polymers such as Poly(methyl methacrylate) (PMMA), amorphous fluorinated polymer (CYTOP), polystyrene (PS) and polycarbonate (PC) for the core, surrounded by a lower-RI fluorinated polymer cladding [7,8,9].

Optical fiber biosensors (OFBs) often utilize the following components in the setup: a light source, an optical fiber with a recognition element, and a detector. They exploit different optical phenomena such as interferometry (Michelson, Mach-Zehnder Interferometer (MZI), Fabry–Perot (F–P), Sagnac interferometers (SI)), surface plasmon resonance (SPR), Surface Enhanced Raman scattering (SERS), and others to achieve ultra-low detection limits and sense very small concentrations of cancer biomarkers present at early stages in oncology patients [10,11].

While optical fibers can employ diverse transduction principles—ranging from SERS [12,13] to fluorescence-based sensors [14]—this review prioritizes RI-based sensors such as interferometers, SPR, localized SPR (LSPR) platforms and Fiber Bragg Gratings (FBG). This choice is driven by the specific advantages of RI sensing, which enables label-free and real-time kinetic measurement of analytes, avoiding the limitations associated with labeling steps, experimental artifacts, or signal bleaching [15]. Furthermore, this study exclusively focuses on silica and polymer optical fibers (POFs) due to their superior practical advantages over chalcogenide and fluoride counterparts. The choice of silica fibers was dictated by their robust surface chemistry (silanol groups) for covalent bioreceptor attachment via silanization, which is more complex to achieve reproducibly on chalcogenide surfaces [16,17,18]. Moreover, silica is chemically inert and stable in the physiological buffers used for cancer cell detection. In contrast, many fluoride glasses are susceptible to degradation in biological materials such as tissue or fluids [19], and chalcogenide fibers often contain heavier toxic elements (As, Sb) that present safety concerns in handling and biocompatibility for further in vivo use. For instance, the potential leaching of arsenic (As) into the surrounding environment can impact negatively on living microorganisms, cells, or tissues. Moreover, another drawback affecting chalcogenide glass fibers is their limited mechanical resistance compared to silicate fibers, which may restrict their robustness for portable or point-of-care clinical applications [20,21].

Optical fibers have demonstrated their potential in the biomedical field by detection of DNA [22,23], protein [24], viruses [25], tumor cells [26,27], etc. Transforming bare optical fibers into biosensors includes surface functionalization, a process of attaching biorecognition elements such as antibodies, aptamers, to the substrate to allow for interaction with target analytes. These biorecognition elements need to be specific to target analytes forming stable interactions while preserving the sensor’s optical properties. Especially in complex media such as serum, achieving good specificity is always a challenge due to non-specific binding and competing biomolecular interactions.

To address these challenges, various techniques have been developed including functionalization such as silanization, polymer coating, layer-by-layer (LbL) assembly, and integration of nanomaterials. The choice of functionalization method greatly affects the sensitivity, selectivity, specificity, stability, reproducibility, and antifouling properties of the optical fiber biosensor [28]. Non-specific binding and surface fouling greatly reduces optical biosensor performance and might lead to false positive results [29]. Consequently, it is crucial to study, develop and enhance current functionalization practices in order to make optical fiber biosensors a reliable and robust tool for early cancer detection.

The progress of fiber-optic biosensors for biomedical applications was extensively studied and summarized in several review articles. Ramola et al. [30] wrote a comprehensive overview of optical biosensing techniques used for cancer research and focusing on sensing mechanisms and device architectures. Meanwhile, Azab et al. [11] in their review article discusses fundamental principles, applications and future perspective of fiber-optic based biosensors in the oncological field. A more recent review article of Kaur et al. [10] concentrated on latest studies utilizing optical biosensors for early detection of cancer highlighting the importance of developing multi-biomarker sensing strategies, and future potential of liquid biopsy approaches and protein markers such as Oct4a. In addition, prior reviews have focused on outlining the various geometries and configurations of optical fiber biosensors, as well as discussing biofunctionalization, dielectric coating strategies and sensing principles such as evanescent wave (EW) and surface plasmon resonance (SPR/LSPR) with reference to plasmonic materials [3,31,32]. The recent SPR-oriented reviews have clearly demonstrated that nanoscale surface engineering of plasmonic interfaces plays an important role in enhancing sensitivity, stability, selectivity and specificity for label-free biomolecular and cancer biomarker detection [33,34].

However, despite these valuable contributions, there has been no work summarizing all surface functionalization steps applied to optical fibers. This review provides a comprehensive overview of various surface functionalization techniques applied to optical fibers for cancer detection. It summarizes and compares modification strategies involving silanization, nanomaterials, polymers, hybrid materials, and molecularly imprinted polymers (MIPs), highlighting their respective performances and limitations.

## 2. Principles and Types of Optical Fiber Biosensors

According to the International Union of Pure and Applied Chemistry (IUPAC), a biosensor is defined as “a device that uses specific biochemical reactions mediated by isolated enzymes, immunosystems, tissues, organelles or whole cells to detect chemical compounds usually by electrical, thermal or optical signals.” OFBs are becoming increasingly pivotal in the biological field, particularly in the domain of oncological diagnostics due to their exceptional sensitivity, compact design, and inherent biocompatibility [35].

In OFBs, light confined within the optical fiber interacts with the surrounding environment through a structurally or chemically modified sensing region, which may be located at the fiber tip or along the fiber length [36]. This interaction enables the conversion of biomolecular binding events occurring at the sensor surface into measurable optical responses. The functionalized fiber structure thus serves as the optical biotransducer, translating biorecognition-induced changes—most commonly variations in the local refractive index—into shifts in wavelength, phase, or intensity of the guided light.

Owing to these characteristics, OFBs are promising tools for the detection of cancer biomarkers, discrimination of cancerous cells, and validation of tumor models in ex vivo and in vivo settings [16]. There are many types of optical fiber biosensors, which are differentiated by two key factors: the choice of optical fiber and the sensing technique used for the detection.

Optical fiber can have single-mode and multimode configurations. Both types show significant promise owing specific fiber architecture and light penetrating mechanisms for cancer detection applications. Single-mode fiber biosensors have demonstrated high sensitivity and versatility for biological detection applications [37]. Their single propagation mode enables precise interferometric and phase-sensitive measurements, making it possible to detect minute refractive index changes associated with biomarker binding. Multimode tilted fiber Bragg gratings (TFBGs) outperform single-mode configuration, especially when biofunctionalized with aptamers targeting HER2 breast cancer biomarkers [35]. Furthermore, hybrid configurations as single-mode-multimode-single-mode (SMS) fiber systems, which use multimode interference techniques, have been widely used to sense proteins and biological compounds [37]. Additionally, polymer or plastic optical fibers (POFs) offer a flexible and cost-effective alternative. For instance, a D-shaped POF SPR biosensor has been developed for the detection of HER2-positive cancer cells with a detection limit of ~5.28 nM and a reaction time of about 5 s [38], showcasing the potential of POF in clinical diagnostics. Beyond material composition, the physical architecture of the fiber itself can be engineered for enhanced performance. For instance, etched multicore fiber (MMF) sensors with plasmonic nanoparticles can sense HepG2, MCF-7, and A549 cancer cells at 2–3 cells/mL [39].

OFB sensing technique involves interferometric, grating-based, and plasmonic methods. Interferometric biosensors measure coherently overlapping waves in the sensing structure. Engineering small gaps or cavities along the fiber creates low-finesse, low-reflectivity mirrors, making the interferometer a compact sensing device. Interferometric sensors with diverse optical designs and sensing algorithms show potential for cancer detection. Traditional interferometers can be improved by switching to micro/nano-scale and microstructured platforms and lowering the fiber diameter to tapered or microfiber [40,41,42,43].

Grating-based fiber optic sensors offer advantages including high precision, compact size, chemical inertness, and multiplexing capabilities for various sensing applications [44]. An FBG is a grating structure formed by inscribing a periodic pattern into the core of an optical fiber, which creates a variation in the refractive index. This structure functions by reflecting a narrow band of light centered at Bragg wavelength. Analyzing the shift in Bragg wavelength allows for the evaluation of refractive index variations in analytes. However, it is worth mentioning that intrinsically FBGs are not sensitive to changes in refractive index as they are not directly exposed to the surrounding medium. Therefore, FBG performance can be enhanced by structurally modifying the fiber, such as through etching or tapering [45], which alters the effective refractive index and causes a measurable spectral shift. Alternatively, sensitivity is improved by using gratings like long-period or tilted Bragg gratings to couple light from the core into the cladding, allowing it to interact with the external environment. These grating-based sensors show significant potential for detecting different cancer types. Various configurations, including tapered fiber interferometers with fiber Bragg gratings [46], long-period gratings [47], and tilted fiber Bragg gratings [48], have demonstrated high sensitivity and specificity for detecting cancer-related proteins. These label-free biosensors can detect biomarkers at concentrations as low as 2 ng/mL [46] and even sub-ng/mL levels [47]. Chai-Chin Chiang et al. [49] created D-shaped long-period fiber grating sensors for gastric cancer detection, demonstrating rapid response within 1 min and high sensitivity of 0.095 nm/μg/mL for G-17 antigen detection.

Plasmonic-based sensors exploit the evanescent wave to excite surface plasmons, coherent electron oscillations at the metal-dielectric boundary. The refractive index changes during biorecognition on a metal-coated fiber surface, changing the resonance state and providing a sensitive detection mechanism [50]. These principles allow label-free biosensing for real-time analysis in compact and versatile setups. SPR and LSPR are effective early cancer detection methods [30].

**Figure 1:**
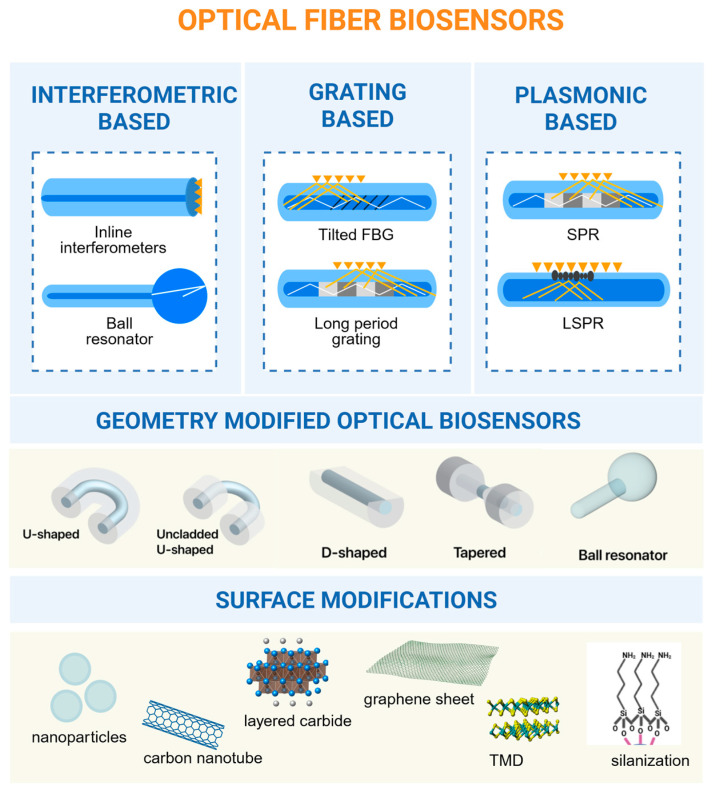
Summary of all optical fiber biosensor types used for the detection of cancer biomarkers as well as surface modification materials.

Interferometric, grating-based, and plasmonic methods are the main sensing methods that make optical fiber biosensors work. Interferometers are great at identifying changes in the refractive index, gratings are great at finding specific biomarkers on small, multiplexed platforms, and plasmonic setups enable analysis of data in real time without using labels and with very quick response times [51]. These approaches are not mutually exclusive; hybrid designs that integrate, for instance, grating structures with plasmonic coatings or interferometric platforms with tapered microfibers have shown synergistic improvements in detection performance [52,53,54]. As fabrication technologies improve and new materials like two-dimensional films and nanostructured coatings become easier to obtain, sensing approaches are likely to move toward highly integrated, small, and clinically useful platforms for finding cancer early. The summarized figure of the types of optical fiber is presented in Figure 1.

## 3. Functionalization Techniques for Optical Fiber Biosensors

**Figure 2:**
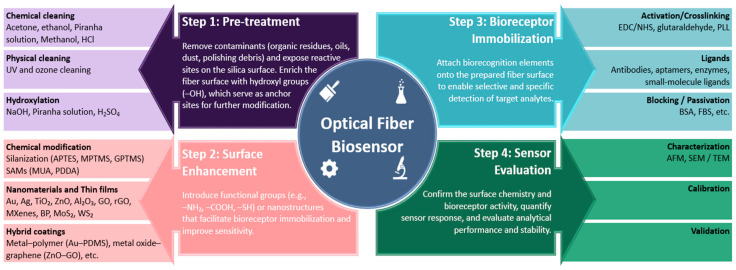
Detailed workflow for surface modification of optical fiber sensors for cancer biomarker detection.

Surface functionalization is an important step in developing optical fiber biosensors, as it directly influences sensing performances such as sensitivity, repeatability, stability, and specificity. The choice of functionalization technique depends on various factors, including the type of optical fiber sensor, the target analyte, and the intended biomedical application. Several commonly used surface functionalization strategies have been developed for the detection of cancer biomarkers. These include silanization, layer-by-layer assembly, nanomaterial coatings, polymer-based modifications, etc. Layer-by-layer technique involves consecutive deposition of functional coatings (polymers, nanomaterials, etc.) on the surface of optical fibers with the ability to closely control the thickness and composition of layers [55]. These techniques demonstrated efficient immobilization of biorecognition elements such as antibodies, aptamers, or peptides onto the surface of optical fibers enabling the detection of cancer proteins and cells with different specificity and low limits of detection. During each step of the fabrication process, the optical fibers are calibrated by evaluating their performance in media with varying RI to verify the success of each functionalization step. The sensor surface can also be characterized by utilizing techniques such as atomic force microscopy (AFM), scanning electron microscopy (SEM), transmission electron microscopy (TEM), fluorescence microscopy, and other techniques [56,57]. The validation of the fabricated sensors is performed by the detection of target analytes in different biological media and comparison of the results with the control samples. Figure 2 below outlines the main steps in developing an optical fiber biosensor, which include pre-treatment, surface enhancement, bioreceptor immobilization, and sensor evaluation.

### 3.1. Pre-Treatment of Optical Fibers

The first step in preparing optical fibers for surface modification and subsequent functionalization is to clean them, removing debris, oils, organic contaminants, and residues. This is typically achieved using methods such as piranha solution treatment, UV-ozone cleaning, or rinsing with ethanol and acetone. The choice of surface cleaning method depends on the intended application and the type of optical fiber used. For instance, while piranha solution is effective for silica fibers, it can damage polymer-clad fibers, making alternative cleaning approaches necessary [58].

Acetone (C_3_H_6_O) is commonly used as an initial step to remove organic contaminants from optical fibers (OFs) before surface functionalization. The fibers are typically immersed in acetone for 20–30 min, optionally with ultrasonic agitation to enhance cleaning efficiency. Following this, the OFs are rinsed with deionized (DI) water and dried. This step is often followed by treatment with either piranha solution or sodium hydroxide (NaOH) to hydroxylate the surface and increase the density of reactive –OH groups. However, acetone cleaning is not a universal practice and is frequently omitted in standard protocols.

The Piranha solution is a mixture of sulfuric acid (H_2_SO_4_) and hydrogen peroxide (H_2_O_2_) used to remove organic residues by strong oxidation at varying ratios, depending on the degree of contamination and the substrate type. Commonly used ratios of H_2_SO_4_ to H_2_O_2_ in a piranha solution are 3:1 [59] and 4:1 [56] for cleaning fiber optic surfaces. Higher ratios such as 7:1 are also mentioned in general chemistry [60], but are not very commonly used for cleaning of optical fibers. Piranha solution not only removes organic matter from the surface but also adds hydroxylic groups (-OH) to the substrate and makes it hydrophilic. Piranha solution should always be freshly prepared, safely handled during the cleaning process, and immediately discarded and not stored for future use [61].

These cleaning procedures are usually combined to not only eliminate unwanted surface particles but also increase the concentration of hydroxyl (–OH) groups on the fiber surface, thereby enhancing its reactivity and preparing it for subsequent functionalization steps such as silanization.

For instance, Kumar et al. [62] utilized a three-step cleaning starting with 20 min in acetone, followed by 30 min in Piranha solution, and then rinsing with ultrapure water. Zhou et al. [63] cleaned fibers by immersing them in acetone for 30 min, and rinsing with DI water. Moreover, in order to increase the number of hydroxyl groups, the fibers were kept in 1.0 M NaOH solution for 1 h. However, cleaning fibers only with Piranha solution [64,65] is a more common practice.

Hydroxylation is an essential step in preparing optical fibers for further functionalization [58]. The main goal of hydroxylation is to enrich the bare optical fiber, intrinsically inert and hydrophobic, with -OH groups. These -OH groups will further serve as reactive sites for silanization, nanomaterial attachment, or direct bioreceptor immobilization [66]. Efficient hydroxylation enables strong and stable attachment of functional layers leading to increased performance and stability of optical biosensors. There are several methods of hydroxylation used such as acid/base treatment, plasma treatment, UV/ozone treatment and others [67].

### 3.2. Surface Enhancement

#### 3.2.1. Silanization

**Figure 3:**
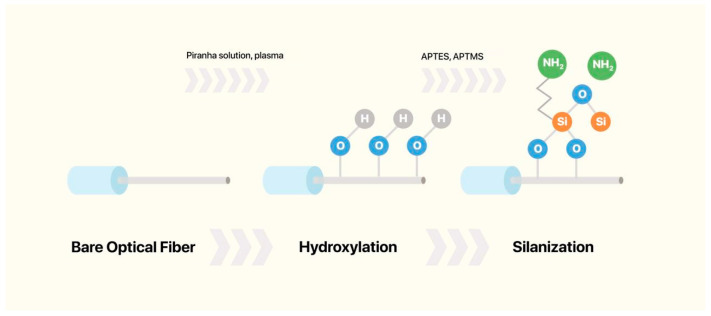
Schematic overview of the functionalization steps up to silanization.

Silanization is a chemical process that involves the covalent bonding of hydroxyl groups (-OH) on a substrate with organosilane compounds to form Si–O–Si bonds, as shown in Figure 3. The main goal of silanization is to increase the hydrophobicity of hydrophilic surfaces, such as glass, metal oxides, and silica [68]. Before silanization, the material surface of interest is thoroughly cleaned to remove all debris and increase the concentration of -OH groups. Silanization of the optical fiber surface enables the covalent attachment of biomolecules for functionalization [28]. Several silane agents can be used for the silanization step, including (3-Aminopropyl)trimethoxysilane (APTMS) [69,70], 3-aminopropyltriethoxysilane (APTES) [53,71], 3-Mercaptopropyl)trimethoxysilane (MPTMS) [72,73,74], and glycidoxypropyltrimethoxysilane (GPTMS) [71].

Murugan et al. [71] discusses two silanization methods: wet-chemical and vapor-phase processes, utilizing silanes such as 3-aminopropyl triethoxysilane (APTES) and 3-glycidyloxypropyl trimethoxysilane (GPTMS) for surface modification of oxide interfaces in biosensor applications.

APTES can be applied at different concentrations depending on the desired surface coverage and biomolecule attachment efficiency. For instance, Nurlankyzy et al. [69] silanized the surface of an SMF (single mode fiber) ball resonator with 1% APTMS in methanol for 20 min. Sypabekova et al. [70] reported a TFBG combined with a ball resonator for ultrasensitive detection of soluble HER2 (sHER2). The glass fiber surface was also functionalized using 1% APTMS. The sensor achieved sensitivities up to 4034 dB/RIU, with LoDs of 151.5 ag/mL in buffer and 3.7 pg/mL in diluted serum, demonstrating both high specificity and remarkable femtomolar detection capability.

In another study, Sun et al. [53] functionalized a tapered optical fiber with HER2 antibodies via silanization of the surface using a 5% (*v*/*v*) APTES solution in ethanol. The resulting biosensor effectively detected HER2 biomarkers in PBS and serum, demonstrating a sensitivity of 0.1 nm/(ng/mL)

Using only the silanization method for functionalizing an optical fiber is cost-effective [75], less time-consuming, and requires fewer operational steps compared to fabricating optical biosensors with nanomaterials or other ‘sandwiched’ assays [76].

After the introduction of reactive functional groups onto the surface of optical fibers via silanization or other activation methods that will be discussed further in this section, crosslinking is used to enable further binding to biorecognition ligands. Commonly used crosslinkers are 1-Ethyl-3-(3-dimethylaminopropyl)carbodiimide (EDC) often used with N-Hydroxysuccinimide (NHS), glutaraldehyde (GA), N,N′-Diisopropylcarbodiimide (DIC) and others [58]. These crosslinkers assist in forming stable covalent bonding between functionalized fiber surface and ligands, thus improving overall sensor stability. More detailed information on crosslinkers will be provided in Section 3.3.

#### 3.2.2. Self-Assembled Monolayers (SAMs)

The self-assembled monolayer (SAM) technique is a surface functionalization method used to create supramolecular architectures by introducing specific functional groups that enable the covalent immobilization of biomolecules such as antibodies and proteins [77]. SAM is known as the most versatile and widely used approach due to low cost, simple manufacturing process, and ability to create uniform functionalized surfaces. It is extensively applied in fiber-optic SPR biosensors.

Zhu et al. [78] built an SPR fiber probe, which upon exposure to bloodstream, can detect CTCs in the blood real-time, specifically MCF-7 breast cancer cells. Authors first deposited a carboxylic SAM using 11-Mercaptoundecanoic acid (MUA), then activated carboxyl groups via EDC/sulfo-NHS, immobilized anti-Epithelial Cell Adhesion Molecule (EpCAM), and blocked the NHS esters with ethanolamine. They reported a sensitivity of 1933.4 nm/RIU, surpassing most reported hetero-core SPR fiber sensors; an LoD of 1.4 cells/mL was achieved after 15 min of on-probe enrichment.

Kim et al. [73] deposited Al sacrificial layer on the multi-mode fiber tip, hydroxylated it and vapor-deposited MPTMS layer. After FIB-patterning the gold nanodisk array, the Al layer was peeled off, leaving gold disks only. Next, a thiol SAM was formed on the fiber surface by 11-MUA, followed by activation with EDC/2-(N-morpholino)ethanesulfonic acid (MES), immobilization of anti-PSA antibody, and blockage with BSA. The sensor showed a linear RI response with an average sensitivity of 5700 RIU^−1^. It detected prostate-specific antigen PSA over 0.1 pg/mL–1 ng/mL in bioassays, achieving a LoD of 1.3 pg/mL.

#### 3.2.3. Nanomaterials-Based

Nanomaterials include materials that have a size between 0.1 and 100 nm in at least one of the three dimensions. Nanomaterials are classified by how many of their dimensions fall within the nanometer scale as 0D (Quantum dots (QDs), fullerenes, and nanoparticles), 1D (nanofibers, nanotubes, nanorods, thin films, and nanowires), and 2D (nanosheets, nanowalls) [79].

0D nanomaterials are particles where all dimensions are nanoscale, giving them spherical or irregular shapes and discrete, delta-function-like density of states, while 1D nanomaterials have one extended dimension and needle-like shapes with density of states that vary as $E^{-\frac{1}{2}}$; 2D nanomaterials are plate-like structures with confinement in one dimension and an energy-independent density of states. These dimensional differences lead to distinct physical and electronic properties of nanomaterials [79,80,81].

**Figure 4:**
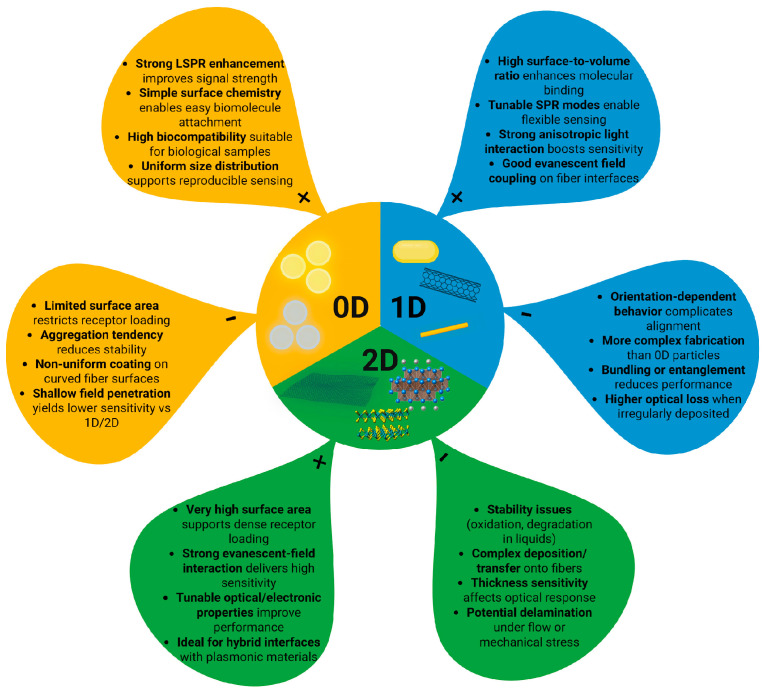
Advantages and Disadvantages of 0D, 1D, and 2D Nanomaterials for fiber-optic biosensing.

Unique optical, chemical, and structural properties of nanomaterials make them particularly attractive for enhancing the performance of fiber-optic biosensors [76]. Figure 4 illustrates this dimensional classification by comparing 0D, 1D, and 2D nanomaterials, showing how their geometry determines their advantages and limitations in fiber-optic biosensing.

**Figure 5:**
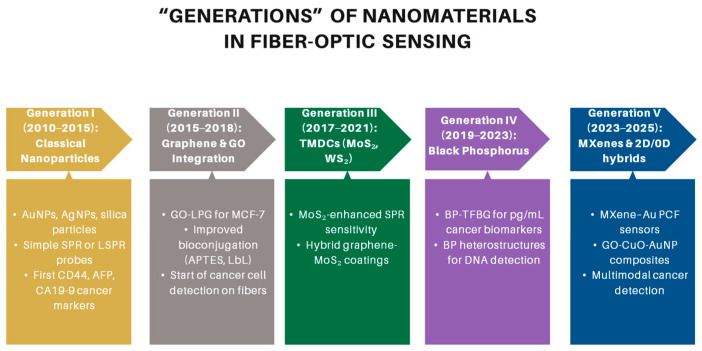
“Generations” of nanomaterials in fiber-optic sensing of cancer biomarkers.

Their integration onto the fiber surface can be achieved through several approaches, including physical deposition techniques such as sputtering or evaporation to generate thin metallic coatings, chemical deposition methods such as layer-by-layer assembly or chemical vapor deposition (CVD) for uniform coverage, and self-assembly strategies that exploit electrostatic interactions or covalent bonding to immobilize nanoparticles [76]. These strategies enable precise tailoring of sensor interfaces, thereby significantly improving sensitivity, selectivity, and stability in cancer detection applications [82,83]. The progressive refinement of these integration strategies has paralleled the broader evolution of nanomaterials used in fiber-optic biosensing, as illustrated in Figure 5.

**0-dimensional nanoparticles-based OFBs.** Gold nanoparticles (AuNPs) are the most extensively used nanomaterials in fiber-optic biosensors, owing to their strong LSPR, high biocompatibility, and versatile surface chemistry that facilitates biomolecule immobilization. Kim et al. [84] demonstrated durable plasmonic fiber sensors fabricated using nanosphere lithography to pattern gold nanostructures. Sensitivity was enhanced through a sandwich immunoassay, with the triangular nanoprobes achieving an ultralow detection limit of 0.25 U/mL for the pancreatic cancer biomarker CA 19-9, which is approximately 700 times lower than the standard reference value. These findings emphasize the reliability and cost-effectiveness of patterned nanostructure-based optrodes for pancreatic cancer detection. In another study, Chen et al. [85] reported a plasmonic TFBG biosensor designed for breast cancer cell detection. The sensor employed an 18° TFBG coated with a 50 nm gold nanofilm and functionalized with GPR30-targeting antibodies. It successfully detected as few as 5 cells/mL within 20 min, exhibited a linear response up to 1000 cells/mL, and showed potential for single-cell detection, making it a simple, compact, and label-free device for real-time circulating tumor cells (CTC) monitoring. Additionally, Zamri et al. [86] developed a fiber-laser biosensor with a gold-coated D-shaped fiber inserted in a single-ring cavity for prostate-cancer biomarker (hCG antigen) detection. Surface functionalization follows a layer-by-layer immobilization technique, starting with gold sputter coating of the fiber surface, followed by sequential adsorption of polyallylamine hydrochloride (PAH), protein G and anti-hCG antibodies. The biosensor demonstrated a sensitivity of 0.2171 nm/(µg/mL) and was capable of detecting hCG concentrations as low as 0.01 µg/mL.

Gupta et al. [82] developed the first portable LSPR-based aptasensor capable of detecting multiple cancer cell lines, including MCF-7 and Caco-2. Their device utilized a U-bent optical fiber that was silanized with APTES, coated with gold nanoparticles, and functionalized with the AS1411 aptamer. Sensitivity tests revealed detection limits of 500 cells/mL in an optical bench setup, with the measured signal intensity ranging from 0.088 to 0.15 a.u., confirming its utility for CTC detection. Similarly, Liang et al. [83] reported a label-free and cost-effective LSPR immunosensor based on a U-bend multimode fiber optic probe for alpha-fetoprotein (AFP) (liver cancer biomarker) detection. To improve nanoparticle adsorption and coating uniformity, the probe surface was pre-treated with microwave-induced H_2_O/Ar plasma, followed by silanization and immobilization of ~70 nm AuNPs. The plasma pretreatment improved refractive index sensitivity and sensor performance, enabling detection in both PBS and human serum across a linear range of 5–200 ng/mL, with limits of detection of 0.85 ng/mL (PBS) and 3.3 ng/mL (serum). This miniaturized probe demonstrates significant potential for clinical biomarker monitoring.

**Figure 6:**
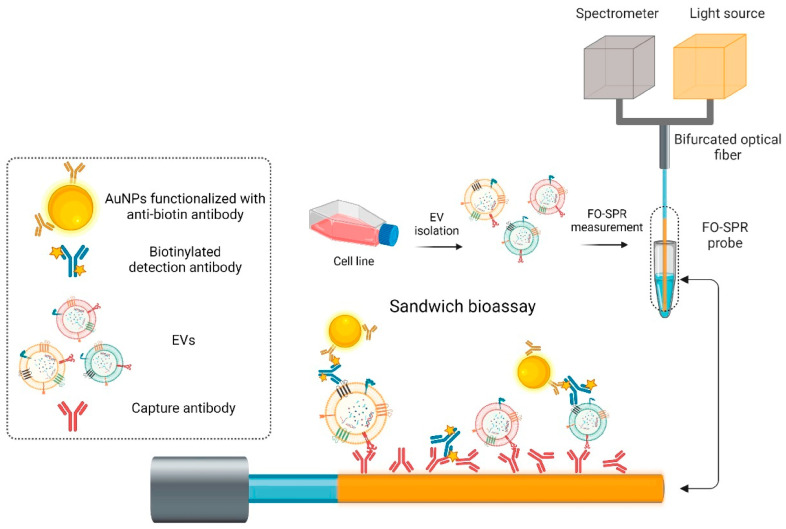
Schematic of the different steps from the fiber optic-SPR EV detection sandwich bioassay. Adapted from [88], licensed under CC BY 4.0.

Building on this concept, Liang et al. [87] also proposed a hetero-core fiber-optic SPR biosensor for the rapid detection of HER2 protein in plasma. The device was fabricated by fusing single-mode and multimode fibers, with a 45 nm gold coating applied to the SMF region to support SPR. Functionalization was achieved through oriented immobilization of anti-HER2 antibodies using thiol-modified protein G, followed by BSA blocking to minimize nonspecific adsorption. Optimizing antibody immobilization improved the sensor’s sensitivity by 2.5-fold, achieving a detection limit of 1 µg/mL under label-free conditions, which demonstrates its potential utility for prognostic breast cancer monitoring. Using the same method of detection, Yildizhan et al. [88] developed a fiber-optic SPR biosensor for label-free detection of extracellular vesicles (EVs) derived from breast cancer cells, which is illustrated in Figure 6. Gold-coated multimode probes were functionalized using COOH-SAM and EDC/NHS chemistry to immobilize anti-HER2 or anti-EpCAM antibodies, followed by a two-step signal amplification using biotinylated detection antibodies (anti-CD9, CD63, CD81) and anti-biotin-conjugated AuNPs. This strategy enabled the specific detection of SK-BR-3 EVs in buffer with a limit of detection (LoD) of 2.1 × 10^7^ particles/mL, while in plasma the LoD was 7 × 10^8^ particles/mL. Similarly, the anti-EpCAM/anti-mix approach allowed detection of MCF7-derived EVs in plasma with a LoD of 1.1 × 10^8^ particles/mL, while maintaining high specificity against healthy donor plasma. This approach demonstrates its promise for liquid biopsy applications in breast cancer diagnostics.

More recently, Wang et al. [89] fabricated an SPR biosensor by magnetron sputtering a gold film onto plastic-clad quartz fibers, followed by covalent immobilization of NMP22 bladder cancer antibodies via 3-MPA and EDC/NHS chemistry. The sensor exhibited strong linearity in the 0–25 ng/mL range with a sensitivity of 0.092 nm/(ng/mL), and its performance was comparable to ELISA in clinical urine samples, underscoring its potential for point-of-care bladder cancer screening.

Alternative nanostructures have also been explored. Ball resonators fabricated through CO_2_ laser splicing of single-mode fibers provide a rapid approach to infrared biosensing [90]. Although their low-finesse spectra are quasi-random and challenging to interpret, the use of the Karhunen-Loève transform (KLT) enabled reliable signal extraction for both refractive index sensing and biomarker detection. Gold-sputtered ball resonators functionalized with anti-CD44 antibodies demonstrated high specificity (30–41%) and an LoD of 19.7 pM, confirming their suitability for cancer biomarker assays despite spectral complexity.

Finally, Lobry et al. [91] introduced an electro-plasmonic biosensor based on gold-coated TFBGs for ultrasensitive detection of HER2 proteins and HER2-positive breast cancer cells. Functionalization was achieved using thiolated HER2-specific aptamers, which enabled selective binding. The biosensor achieved a detection limit of 8.36 fM for HER2 protein and 1000 cells/mL for HER2-positive cells, supporting real-time and label-free detection in small sample volumes.

In addition to these conventional approaches, environmentally friendly synthesis routes have also been investigated [92]. An optical fiber ball resonator biosensor functionalized with AuNPs synthesized using green tea extract was reported for the detection of the CD44 biomarker. This eco-friendly strategy not only reduced chemical toxicity during nanoparticle preparation but also provided excellent sensing performance, with an intensity change of approximately 13.17 dB across a concentration range from ~42.9 aM to 100 nM, and a limit of detection of 0.111 pM.

Beyond gold, other metal and metal-oxide nanoparticles have been incorporated into fiber-optic biosensors to improve sensitivity, specificity, and functionality, often by providing complementary optical properties or enhanced bio-interfaces [93].

One example is the use of copper oxide nanoflowers (CuO-NFs) combined with gold nanoparticles and graphene oxide on an etched multicore fiber (MCF) platform [39]. Singh et al. developed a label-free LSPR sensor by etching a seven-core MCF to enhance evanescent field interaction. The surface was functionalized with an optimized size of AuNPs for plasmonic amplification, graphene oxide (GO) for improved biocompatibility, and CuO nanoflowers to assist in binding and signal enhancement. A further functionalization with 2-deoxy-D-glucose (2-DG) enabled targeting of cancer cells. They demonstrated detection limits of 2–3 cells/mL for several cancer cell lines (MCF-7, HepG2, etc.) over a linear range of 10^2^ to 10^6^ cells/mL [39].

**Figure 7:**
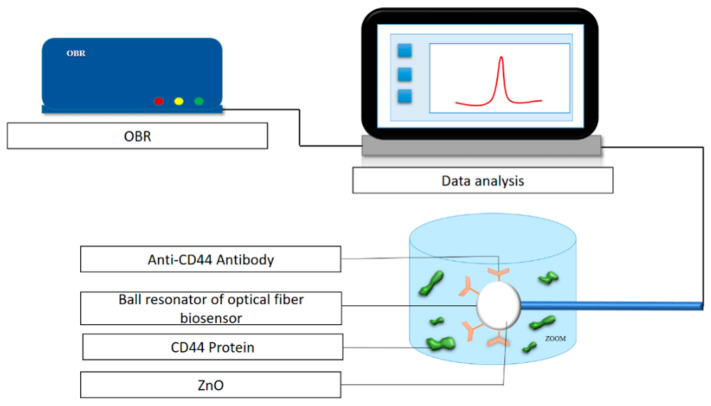
Experimental setup for the detection of the CD44 protein using a biofunctionalized sensor. Adapted from [64], licensed under CC BY 4.0.

Zinc oxide (ZnO) nanoparticles have also been studied by Paltusheva et al. [64]. A study developed a spherical-tip fiber-optic sensor coated with ~100 nm ZnO (via sol–gel deposition) to detect CD44 protein (a cancer metastasis marker). The sensor showed a detection limit as low as 0.8 fM and was capable of regenerating the sensing surface without damage. The schematic of experimental configuration is shown in Figure 7.

**1-dimensional nanoparticles-based OFBs (nanowires, nanorods, nanotubes).** While 0D nanoparticles provide strong localized plasmonic effects, 1D nanostructures—including nanowires, nanorods, and nanotubes—introduce additional advantages in optical biosensing due to their high aspect ratio, anisotropy, and enhanced surface-to-volume ratio [94]. These features result in stronger light-matter interactions, guided plasmon propagation, and more efficient biomolecule immobilization, which can significantly improve the sensitivity of fiber-optic biosensors.

For example, gold nanorods (AuNRs) exhibit two distinct SPR modes (longitudinal and transverse), enabling tunability of optical response across the visible to near-infrared (NIR) spectrum, which is particularly useful for biosensing in biological transparency windows [95]. Building on this property, Liang et al. developed an AuNR-black phosphorous nanointerface on an optical microfiber that achieved ultrasensitive HER2 detection down to 0.66 aM and simultaneously enabled NIR-triggered photothermal/chemotherapy, demonstrating the theranostic potential of AuNR-based platforms [95].

Li et al. presented a micro-tapered long-period grating on single-mode fiber that uses gold nanorods to detect an alpha-fetoprotein (AFP), a hepatocellular carcinoma marker [74]. The surface is silanized with MPTMS to anchor HS-PEG-COOH-modified gold nanorods, then carboxyls are EDC/NHS-activated to covalently immobilize anti-AFP monoclonal antibodies. The sensor showed a linear assay range of 10–400 ng/mL with sensitivity 3 pm/(ng·mL^−1^) and LoD equal to 17.6 ng/mL [74].

**2-dimensional nanoparticles-based OFBs (nanosheets, graphene oxide (GO), molybdenum disulfide (MoS2), and MXenes).** Two-dimensional (2D) nanomaterials such as graphene, molybdenum disulfide (MoS_2_), MXenes, and black phosphorus (BP) have emerged as exceptional candidates for enhancing the sensitivity, selectivity, and biocompatibility of optical fiber biosensors for cancer detection. Their high surface-to-volume ratio, tunable electronic properties, and excellent light-matter interaction enable improved immobilization of bioreceptors and enhanced transduction of biomolecular binding events into optical signals [96].

Graphene and its derivatives (e.g., graphene oxide, GO; reduced graphene oxide, rGO) have been the most extensively studied 2D coatings for fiber-based biosensors due to their outstanding electrical conductivity, broadband optical absorption, and functionalization versatility. Graphene layers on optical fiber platforms can increase surface plasmon coupling and improve evanescent field penetration, thereby boosting sensitivity for detecting cancer biomarkers [39,84,97,98,99,100].

Several studies have explored graphene-coated fiber-optic SPR sensors targeting cancer-related biomarkers. Yang et al. developed a graphene-silver (Gr/Ag) coated TFBG sensor that achieved enhanced refractometric response and potential applicability for detecting cancer biomarkers in complex media [97]. Recent reviews by Zhang et al. summarized advances in graphene-based optical fiber biochemical sensors, highlighting their role in biosensing platforms through integration strategies such as Langmuir-Blodgett deposition, chemical vapor deposition (CVD), and solution-phase transfer [101].

In the previously mentioned studies graphene oxide was used in complex structures together with gold and copper oxide for oncological detection [39,84,97,98]. Furthermore, Sun et al. [99] experimentally demonstrated a graphene oxide-functionalized long-period fiber grating biosensor for label-free detection of MCF-7 breast cancer cells, where GO nanocoatings were deposited via an in situ layer-by-layer assembly method, enabling indirect cell density sensing through metabolite-induced refractive index changes with a detection limit of 270 cells·mL^−1^. In parallel, Deng et al. experimentally demonstrated a graphene oxide-functionalized microfiber biosensor for label-free detection of alpha-fetoprotein, a key biomarker for early hepatocellular carcinoma. The GO coating, immobilized via APTES-assisted silanization and EDC/NHS antibody coupling, enabled ultra-sensitive AFP detection with a sensitivity of 1.12 nm per logarithmic concentration change and a detection limit of 78 zg·mL^−1^ [100].

Notably, graphene oxide is preferred over pure graphene in cancer biosensing. The reason behind it is that an oxygen-rich surface enables facile bioreceptor immobilization, aqueous processability, reduced nonspecific adsorption, and effective integration with plasmonic nanostructures [102]. All of these points are critical for sensitive and reliable cancer biomarker detection.

The alternative 2-dimensional MoS_2_, a member of the transition metal dichalcogenide (TMDC) family, offers a direct band gap and large optical absorbance in the visible and near-infrared regions, making it particularly suitable for optical sensing. Its layered structure facilitates biofunctionalization and supports strong evanescent field enhancement when integrated with optical fibers [103]. Qin et al. reviewed the use of MoS_2_ in optical and electrochemical biosensing of cancer biomarkers, summarizing multiple platforms achieving picomolar-to-femtomolar detection limits for analytes such as carcinoembryonic antigen (CEA) and prostate-specific antigen (PSA) [103]. Although most reported MoS_2_-based biosensors operate in electrochemical or photoluminescent modes [104,105], their integration with optical fibers—especially SPR and interferometric configurations—has shown theoretical promise (mentioned in Section 4) [106,107].

MXenes, another class of 2D transition metal carbides and nitrides, have recently attracted significant attention as plasmonically active and biocompatible coatings for fiber-optic biosensors. Their metallic conductivity and tunable surface terminations (-OH, -O, -F) enable efficient coupling with evanescent fields and stable biofunctionalization. Experimental [108] and theoretical [109,110] works proves that MXenes in combination with gold can be potentially used for oncological detection.

In parallel to these approaches, black phosphorus (BP) has emerged as another promising 2D nanomaterial due to its anisotropic electronic properties, tunable band gap, and strong near-infrared absorption. In contrast to graphene and MoS_2_, BP offers higher photoresponsivity and intrinsic semiconducting behavior, making it advantageous for biosensing in the biological transparency window [96]. Zhou et al. [63] reported one of the first experimental demonstrations of a BP-functionalized tilted fiber grating (TFG) biosensor for detecting neuron-specific enolase (NSE), a cancer-related biomarker, achieving a detection limit near 1 pg/mL.

Among all 2D nanomaterials, GO- and BP-based fiber sensors have shown the most direct experimental validation for cancer biomarker detection [39,63,84,95,97,98,99,100], while current studies with MoS_2_ and Mxene are mostly focused on simulations [106,107,109,110].

#### 3.2.4. Hybrid Structure-Based OFBs

Hybrid organic–inorganic nanostructures are engineered interfacial architectures in which organic components (e.g., polymers, graphene derivatives) are chemically or physically integrated with inorganic or metallic materials (e.g., Au, Ag, metal oxides) to form a single functional sensing layer on the optical fiber surface. In OFBs, such hybrid structures are designed to combine the optical and plasmonic properties of inorganic or metallic nanomaterials with the chemical functionality, flexibility, and biocompatibility of organic layers, resulting in highly stable, high-surface-area, and bioactive interfaces [27,39,98,108,111]. These interfaces facilitate efficient bioreceptor immobilization, suppress nonspecific adsorption, and enhance light-matter interactions, thereby enabling sensitive and selective detection of cancer-related biomarkers, including cells, proteins, nucleic acids, and extracellular vesicles.

**Figure 8:**
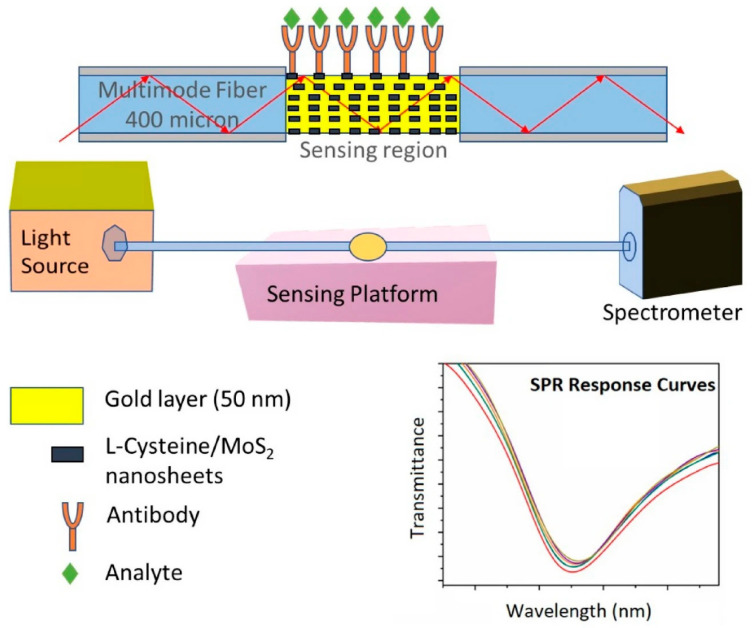
Experimental setup of the MoS_2_ modified OF-SPR biosensor. Adapted from [111], licensed under CC BY 4.0.

Li et al. [108] developed an optical microfiber biosensor integrated with Ti_3_C_2_-supported gold nanorod hybrid interfaces for ultrasensitive detection of renal cancer biomarker CAIX and live cancer cells. Exploiting strong evanescent field coupling, the sensor achieved detection limits as low as 13.8 zM (buffer), 0.19 aM (serum), and 180 cells/mL in culture media, enabling precise early-stage renal cancer diagnosis. In another study, Thawany et al. [111] developed a fiber SPR sensor by depositing an L-cysteine/MoS_2_ composite on a gold-coated D-shaped fiber, which is shown in Figure 8. The MoS_2_ nanosheet layer formed a robust interface for antibody binding. In testing, the sensor quantitatively detected proteins ferritin and IgG (general biomarkers elevated in some types of cancer), with linear ranges 50–400 ng/mL and LODs of 12 ng/mL (ferritin) and 7.2 µg/mL (IgG).

Extending these concepts, Zu et al. [27] developed a nanotube-functionalized and microfluidic-integrated multiresonance optical fiber biosensor for rapid and highly sensitive tumor cell detection. A TFBG was inscribed into a standard SMF-28 fiber and functionalized with a uniform, self-assembled coating of halloysite nanotubes (HNTs), arranged via vertical evaporation. This slit-like patterned HNT layer enhanced cell capture efficiency and light scattering sensitivity. The biosensor was tested on various cancer cell types including MCF-7, MDA-MB-231, 4T1, and MGC-803, and showed clear discrimination between tumor and normal cells within minutes. Integrated with a microfluidic chip, the sensor enabled low-volume, real-time analysis with a limit of detection of 10 cells/mL and a linear response in the 10–10^5^ cells/mL range.

Following this line of developments, Singh et al. [39] developed a compact, portable, label-free fiber optic LSPR biosensor by splicing a seven-core Multi-Core Fiber (MCF) with a Single-Mode Fiber (SMF) to form an etched SMF–MCF probe. The etched structure enhanced evanescent wave interaction and modal coupling. The fiber surface was functionalized via silanization with ethanol-based silane, followed by sequential immobilization of GO, gold nanoparticles (AuNPs), and copper oxide nanoflowers (CuO-NFs). This nanomaterial combination improved LSPR sensitivity and biocompatibility. The sensor was further coated with 2-deoxy-D-glucose (2-DG) to selectively target GLUT receptor-overexpressing cancer cells. The probe demonstrated ultra-sensitive detection of various cancer cell lines including HepG2, Hepa1–6, A549, and MCF-7, achieving a limit of detection (LoD) as low as 2 cells/mL and a broad linear detection range of 10^2^–10^6^ cells/mL [39]. Combination of gold and graphene oxide was also used by Qiu et al. [98] to build Fabry–Perot interferometer (FPI) biosensor based on a D-shaped hollow-core photonic crystal fiber (HCPCF) for ultrasensitive detection of progastrin-releasing peptide (ProGRP). The fiber surface was functionalized with a graphene oxide layer and followed by in situ growth of gold nanoparticles (AuNPs) to create a stable AuNP@GO nanointerface [98]. This hybrid 2D/3D coating enabled high biomolecule loading and strong plasmonic coupling, yielding a high liquid refractive index sensitivity of 583,000 nm/RIU and a detection limit of 17.1 ag/mL, corresponding to the single-molecule level.

These advances illustrate how hybrid nanomaterials do not simply add functionality to optical fibers but redefine the sensing interface itself, creating a synergistic environment where chemical affinity, optical confinement, and nanostructured geometry collectively elevate the precision and early diagnostic capability of fiber-optic biosensors.

#### 3.2.5. Polymer-Based OFBs

Polymer materials can also be applied to modify the surface of optical fibers, improving analyte binding and enabling signal amplification. Depending on the design, polymers may form the fiber substrate itself or act as active interfacial layers. Immobilization methods include dip-coating, in which optical fibers are immersed in polymer solutions to form thin films; electrospinning, which produces nanofibrous polymer layers with increased surface area; and polymer grafting, involving the covalent attachment of polymers to the fiber surface via silanes or thiols [84,112,113,114,115,116,117,118].

One of the reasons for using polymer optical fibers is their ability to enhance sensitivity due to the material’s low RI. Cennamo et al. [112] developed SPR-based D-shaped polymer optical fiber biosensor for the early detection of HER2-positive breast cancer. The side-polished CYTOP fiber (core/cladding diameter 120/490 μm, residual thickness 245 μm) was coated with a 50 nm gold layer via vacuum magnetron sputtering. The gold surface was then functionalized using a self-assembled monolayer of MUA, activated with EDC/NHS chemistry, and immobilized with HER2-specific aptamers, followed by BSA blocking to reduce nonspecific adsorption. The biosensor exhibited a sensitivity of ~1.37 nm shift at 1 μg/mL HER2 (5.5 nM), with a limit of detection of ~5.28 nM and a rapid response time of 5 s.

Polymers may serve as fabrication tools as well. Kim et al. [84] in their study utilized top-down nanosphere lithography (NSL) to fabricate gold nanostructures on multimode optical fiber surfaces for detecting carbohydrate antigen 19-9 (CA19-9), a pancreatic cancer biomarker. The surface functionalization involved self-assembly of polystyrene (PS) beads, gold deposition and lift-off techniques, followed by modification with 11-mercaptoundecanoic acid (MUA) for antibody attachment. In this approach, PS self-assembling colloidal nanospheres served as a sacrificial template, guiding the geometry of resulting metallic nanostructures. The biosensor achieved a LoD of 0.25 U/mL.

In another study, Li et al. [113] investigated the leverage of the pH-responsive RI behavior of polyacrylic acid (PAA)/chitosan (CS) composite, which allows more precise DNA hybridization detection. Authors developed a dual-region optical fiber biosensor combining FBG and SPR for detecting the EGFR exon-20 gene, associated with lung cancer. Apart from the PAA/CS coating, the fiber surface was functionalized with MUA, EDC/NHS, PEI, and probe DNA for hybridization detection. This design enabled simultaneous monitoring of DNA, temperature and pH, achieving a detection limit of 13.5 nM. Similarly, Kong et al. [114] developed a Ω-shaped fiber optic LSPR biosensor for CTC detection, specifically focusing on MCF-7 breast cancer cells. They first coated the fiber surface with polydopamine (PDA) through the self-polymerization process of dopamine. Due to the abundance of amino groups on the PDA coating, it acted as an adhesion layer that anchored gold nanoparticles (AuNP) and gold nanorods (AuNR) onto the fiber surface through electrostatic interactions. The hybridized nanolayer was further modified with MUC1 aptamers, which selectively captured MCF-7 cells. Authors could achieve a RI sensitivity of 37.59 a.u/RIU.

Wei et al. [115] employed an optical microfiber coupler (OMC) immunosensor to quantitatively detect the carcinoembryonic antigen (CEA) in human serum. Surface functionalization was based on LbL electrostatic assembly of poly(diallyldimethylammonium chloride) (PDDA) and poly(acrylic acid) (PAA). Alternating positively charged PDDA and negatively charged PAA layers generated a stable charged interface, while PAA provided carboxyl groups that were subsequently activated with NHS/EDC to enable covalent immobilization of anti-CEA antibodies. Then, residual sites were blocked using ethanolamine and preadsorbed serum to reduce nonspecific adsorption. This biosensor demonstrated ultrahigh sensitivity based on the interference turning-point effect, achieving a detection limit as low as 34.6 fg/mL (0.475 fM) with a wide dynamic range. On the other hand, for the detection of CEA, Li et al. [116] modified the surface of TFBG optical fiber using an electrospun polyacrylonitrile (PAN) nanofiber membrane and gold nanomembrane. The PAN nanofiber layer provided larger surface area and porosity, resulting in immobilization of more anti-CEA antibodies. Additionally, the PAN layer acted as a biocompatible bridge between the inorganic fiber surface and the organic biorecognition elements. The PAN-based biosensor demonstrated a sensitivity of 0.46 dB/(µg/mL) and a low limit of detection of 505.4 ng/mL in buffer.

Likewise, Xiao et al. [117] presented a tapered optical microfiber biosensor functionalized with a polystyrene (PS) and gold nanosphere interface for detecting CEACAM5, a biomarker associated with gastrointestinal, colorectal, and pancreatic cancers. The PS and gold nanospheres provided a synergistic sensitization effect: the PS core increased the effective surface area, thus facilitating antibody immobilization, while the gold nanospheres amplified the localized electric field intensity. The surface functionalization involved gold nanoparticle immobilization, antibody functionalization via glutaraldehyde crosslinking, and electrostatic assembly on the microfiber. The biosensor achieved a bulk refractive index sensitivity of 0.86 nm/lg M with a limit of detection (LoD) of 3.54 × 10^−17^ M in pure solution and 5.27 × 10^−16^ M in serum. This approach significantly improves point-of-care testing (POCT) capabilities for ultralow biomarker concentrations.

In addition to the studies discussed above, Hu et al. [118] developed an antibody-modified magnetic microsphere (MMS) biosensor designed for CEACAM5 protein detection in human serum. The biosensor uses optical fiber with a peanut structure cascaded lasso (PSCL) design to enhance sensitivity. The surface functionalization method involves piranha solution treatment, APTES silanization, glutaraldehyde crosslinking, and antibody immobilization with Fe_3_O_4_-coated polystyrene MMS, followed by BSA blocking to prevent non-specific binding. The polystyrene MMS were introduced to form a sandwich structure (“antibody–antigen–antibody-MMS”), which amplified the immune binding signal by increasing the RI variation near the fiber surface, resulting in an ultra-low limit of detection (LoD). The study reports a LoD of 0.11 ng/mL, which meets clinical screening thresholds [118].

Taken together, these papers demonstrate that surface modification and nanomaterial functionalization enable optical signal enhancement, while the biorecognition elements—typically an antibody, aptamer, nucleic acid probe, etc., provide molecular recognition and target specificity. Following surface activation and functional layer formation, these bioreceptors are immobilized onto the modified fiber surface using covalent, electrostatic, or affinity-based approaches, thereby completing the biosensing interface. Table 1 summarizes all surface functionalization strategies applied to optical fibers for cancer biomarker detection.

### 3.3. Biorecognition Elements and Surface Passivation Strategies

Biorecognition elements are molecular components responsible for the specific interaction with a target analyte, forming the recognition layer of a biosensor. They include affinity-based biological receptors such as antibodies, aptamers and nucleic acid probes, which rely on specific biochemical interactions, as well as synthetic recognition elements such as molecularly imprinted polymers (MIPs), where specificity arises from structurally complementary binding sites [123].

One of the most important steps in fabrication of optical fiber biosensors is the process of immobilization of biorecognition elements of the optical surface. If the procedure is done correctly, the resulting biosensor will form stable attachment to target analytes with great reproducibility and reliability. Crosslinking plays an essential role in forming covalent bonds between functionalized surfaces and biomolecules. One of the most commonly used crosslinkers is glutaraldehyde [24,117,118]. GA is a bifunctional aldehyde crosslinker: one aldehyde group reacts with amines on the optical surface, for example after silanization, and the other group bonds with amines located on biomolecules such as antibodies, aptamers, nuclei acids. The reaction chemistry of GA is rather complex and requires careful control to achieve desired results [124].

EDC in combination with NHS [74,88,89,112,113] is another widely used water-soluble crosslinker in preparation of optical fiber biosensors. EDC activates carboxyl groups, while NHS acts as a helper forming more stable NHS esters, which later react with amine groups on biorecognition elements. EDC/NHS crosslinking approach is recognized for its great efficiency and biocompatibility in many biomedical applications [125].

#### 3.3.1. Biorecognition Elements (Antibodies, Aptamers, Nucleic Acids, Metabolic Analogs, Biotin, MIPs)

Antibodies remain the most widely used bioreceptors in optical fiber biosensors for cancer biomarkers such as CD44, HER2, CEACAM5, PSA, and CA-125. Bekmurzayeva et al. [56] reported a spherical fiber-tip biosensor for CD44 protein, where the silica tip was silanized with APTMS, coated with a thin gold film, modified with a MUA, and then activated with EDC/NHS to covalently immobilize anti-CD44 antibodies; subsequent blocking with bovine serum albumin (BSA) yielded a picomolar-level limit of detection in buffer and serum. Similar silanization-plus-antibody protocols were later applied to shallow-tapered optical fibers for rapid CD44 detection in the tens of fM-nM range, highlighting the robustness of this surface chemistry for label-free cancer sensing [120]. Expanding upon previous CD44 protein sensors Nurlankyzy et al. [69] and Myrkhiyeva et al. [121] extended the studies for dynamic CD44 measurements in complicated media and label-free identification of CD44-expressing breast cancer cells, highlighting the significance of stable, targeted antibody layers for in situ cancer diagnostics.

Aptamers are short single-stranded DNA or RNA sequences that offer precise molecular recognition while avoiding batch-to-batch variability and denaturation issues commonly associated with protein-based receptors [126]. In fiber-optic cancer biosensors, aptamers have been immobilized through thiol-gold chemistry or silane-based coupling to target biomarkers such as HER-2, anti-nucleotin, CAIX, MUC1 [38,82,91,108,114]. HER2-targeting aptamers have been successfully employed as robust biorecognition elements in plasmonic fiber-optic biosensors for breast cancer detection. In an early demonstration, a D-shaped gold-coated polymer optical fiber SPR biosensor functionalized with HER2 aptamers enabled rapid, label-free detection of HER2 protein, achieving a response time of ~5 s and a limit of detection of ~5.3 nM, highlighting its potential for early serum-based diagnostics [38]. Building on this approach, an electro-plasmonic SPR-TFBG biosensor integrated electrophoretic preconcentration with aptamer-based recognition, allowing active attraction of HER2 proteins and HER2^+^ cells toward the evanescent field prior to binding [91].

DNA probes are nucleic-acid based biorecognition elements, and unlike aptamers, which rely on three-dimensional affinity binding to proteins or cells, DNA probes target specific genetic cancer biomarkers such as oncogenic mutations, microRNA signatures, and circulating DNA [127]. In interferometric platforms, probe DNA immobilized on an exposed-core microstructured optical fiber enabled real-time detection of complementary DNA via refractive index modulation at the evanescent field, achieving a nanomolar range sensitivity and limit of detection [22]. In a complementary plasmonic–grating approach, probe DNA functionalized on a dual-SPR Fiber Bragg grating allowed selective detection of the EGFR exon-20 lung cancer gene [113].

In contrast to affinity-based recognition, metabolic analogs exploit the altered metabolic phenotype of cancer cells as the sensing mechanism [39,128,129]. Cancer cells exhibit elevated glucose uptake due to the overexpression of glucose transporters (GLUTs) and enhanced glycolytic flux (Warburg effect) [128]. Metabolic analogs such as 2-deoxy-D-glucose (2-DG) are preferentially transported into cancer cells via GLUTs and become intracellularly trapped after phosphorylation [129]. When incorporated into optical fiber biosensors, 2-DG enables selective targeting of metabolically active tumor cells, leading to measurable optical changes. This metabolism-driven recognition strategy has been applied for the detection of small-cell lung cancer-associated biomarkers, including neuron-specific enolase (NSE) and pro-gastrin-releasing peptide (ProGRP) [39].

Beyond biologically mediated recognition strategies, specificity in optical fiber biosensors can also be achieved through entirely synthetic approaches. MIPs immobilized onto the surface of the sensing device can serve as a highly sensitive and selective recognition element for target analytes [130]. Synthetic, biomimetic MIPs are made by polymerizing functional monomers and cross-linkers around a target molecule (template). When the template is removed, the MIPs form highly specific recognition sites that resemble natural receptors, providing strong stability, durability, and selectivity for analyte detection even in harsh environments [131]. According to review made by Yang et al. [132] molecularly imprinted polymers have been successfully integrated with multiple optical fiber sensing architectures, including fluorescence-based probes, SPR and LSPR fiber sensors, grating-based sensors (LPGs and TFBGs), interferometric configurations, lossy-mode resonance sensors, and intensity-distribution platforms. These MIP-functionalized fiber sensors were evaluated in diverse media ranging from aqueous solutions to complex biological fluids such as serum, plasma, urine, saliva, and cerebrospinal fluid, as well as food samples and gaseous environments. Although the reviewed MIP-functionalized optical fiber sensors were not specifically tested for cancer biomarkers, their successful operation in complex biological media and proven capability for selective protein detection highlight their strong potential for future label-free cancer diagnostics.

**Figure 9:**
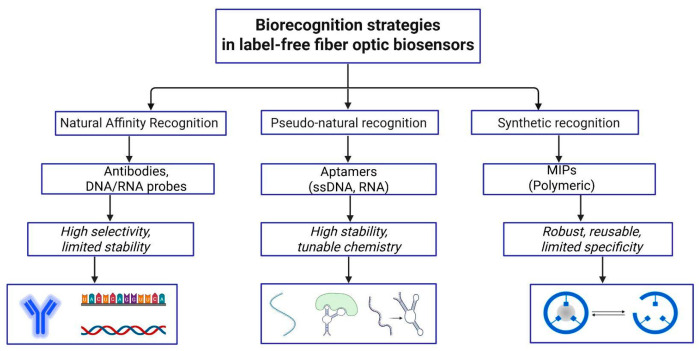
Biorecognition strategies in label-free fiber optic biosensors.

Figure 9 below summarizes all biorecognition elements highlighting their characteristics in the context of their use for fiber optic sensors.

#### 3.3.2. Blocking and Anti-Fouling Strategies

For cancer biomarker detection in complex biological media, such as blood, serum, plasma, urine, high molecular specificity alone is insufficient to ensure reliable performance. Following bioreceptor immobilization, non-reacted active sites and exposed surface domains can promote nonspecific adsorption, leading to background signal drift and compromised selectivity [133]. Therefore, blocking and anti-fouling are used to prevent these problems. Blocking ensures local passivation of residual binding sites near the bioreceptor [134], whereas anti-fouling coatings provide global resistance to nonspecific adsorption [133] and are crucial for maintaining selectivity in complex biological environments.

Common blocking agents for fiber optic biosensors, such as bovine serum albumin (BSA), PEG, Tween-20, ethanolamine occupy unreacted functional groups and hydrophobic patches without interfering with target binding.

BSA is employed to block the unreacted hydrophobic or charged patches on silane, SAM, or polymer-modified fiber surfaces at a concentration of 0.1–1% (*w*/*v*) in buffer. The use of BSA reduces the non-specific binding as it was confirmed by atomic force microscopy and control assays [56].

Poly(ethylene glycol) (PEG) and PEG-containing copolymers can be adsorbed onto the surface of optical fiber biosensors after antibody immobilization to create hydration layers and provide steric repulsion that significantly prevent non-specific protein adsorption. To achieve nearly total background signal suppression in complicated biological fluids, PEG or PEGylated SAMs are frequently used with BSA in biosensing workflows [57,92].

To break weak hydrophobic contacts, non-ionic surfactants like Tween-20 are often added to blocking or rinsing solutions. To get the best blocking performance without upsetting the bioreceptor layer, several procedures mix BSA, PEG, and low quantities of Tween-20 [57,92].

Ethanolamine is commonly used as a post-immobilization blocking agent in EDC/NHS-based functionalization schemes to deactivate residual activated carboxyl groups [135]. In fiber optic LSPR sensors based on CM-dextran–modified gold nanoparticles, ethanolamine (0.5–1.0 M, pH ≈8.0–8.5) is applied after antibody immobilization to quench residual NHS esters and reduce local nonspecific adsorption [136]. A similar approach is used in lab-on-fiber and SPR gold-chip biosensors, where 1 M ethanolamine-HCl (pH 8.5) deactivates unreacted carboxyl groups following EDC/NHS coupling, stabilizing the sensor baseline [137].

Blocking therefore contributes to local selectivity by minimizing false binding events near the sensing interface, but it does not provide long-term protection against fouling in complex biological fluids. In realistic bioanalytical conditions, selectivity must be understood as the ability of the sensing interface to resist nonspecific adsorption from complex media such as serum, plasma, or cell culture supernatants while preserving the accessibility of the target-specific biorecognition sites [29,58]. This requirement is addressed by anti-fouling strategies, which introduce highly hydrated and chemically inert surface layers that suppress nonspecific protein and cell adhesion over extended operation times.

Antifouling strategies reported in the literature primarily rely on hydrophilic and zwitterionic interfaces, implemented via self-assembled monolayers and polymer brushes. Notably, PEG can act either as a short-range blocking component when sparsely incorporated [57,92] or as a true anti-fouling layer when organized into dense, well-hydrated monolayers or polymer brushes [138]. In [138] PEG incorporated as dense polymer brush grown directly from the gold surface, rather than used as a terminal blocking layer. In this protocol, the gold-coated fiber is first modified with an alkanethiol initiator SAM, followed by surface-initiated polymerization to form a ~100 nm thick, highly hydrated PEG-derived brush.

In parallel, antifouling peptide monolayers have been implemented on gold-coated fiber-optic SPR probes by forming mixed self-assembled monolayers of zwitterionic peptides and biorecognition elements [139]. In these approaches, cysteine-terminated peptides with alternating charged residues were co-immobilized with aptamers via Au-S bonding, creating a dense, charge-neutral, highly hydrated monolayer that suppresses nonspecific protein adsorption. The peptide SAM acts as a hydration-driven, sterically repulsive interface, enabling reliable detection in complex media such as 10% human serum [139].

Hydrophilic hydrogels, including polysaccharide-based materials, such as dextran hydrogel, along with synthetic materials like PEG and zwitterionic materials, provide strong antifouling layers that mitigate protein and cell adhesion on biosensor surfaces [140]. For example, PEGylated gold surfaces coated with dextran hydrogel provided surface immobilization and detection limit in SPR-type sensors, while maintaining low fouling in complex matrices [141]. In another study, carboxymethyl dextran (CMD) as a multifunctional layer for gold nanoparticles and as an interfacial layer on the fiber surface. CMD simultaneously acted as a mild reducing agent, a steric stabilizer, and an antifouling polymer, while providing surface carboxyl groups for EDC/NHS-mediated conjugation of DNA probes. It led to ultrasensitive ssDNA detection at the femtomolar level without amplification [142].

**Figure 10:**
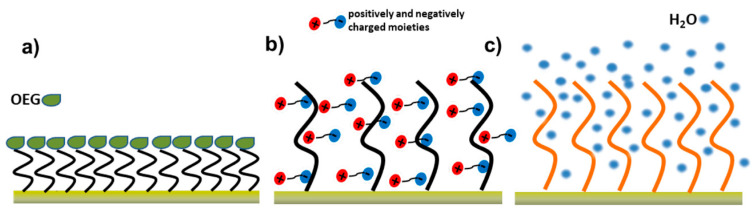
Schematic representation of representative antifouling interfaces: (**a**) self-assembled monolayers functionalized with oligo(ethylene glycol) (OEG) chains, (**b**) zwitterionic coatings, and (**c**) hydrogel-based polymer layers. Adapted from [140], licensed under CC BY 4.0.

These approaches represent distinct architectural realizations of hydrophilic and zwitterionic antifouling interfaces, which can be broadly classified into monolayer-based, brush-based, and hydrogel-based designs. Figure 10 below generalizes all anti-fouling strategies.

For optical fiber cancer biosensors that work directly in serum or cell culture media, where the sensor must distinguish low-abundance target biomarkers from a significant excess of non-specific proteins and cells, properly adjusted blocking is particularly important [56,57,92,135,137,139,141,142].

**Figure 11:**
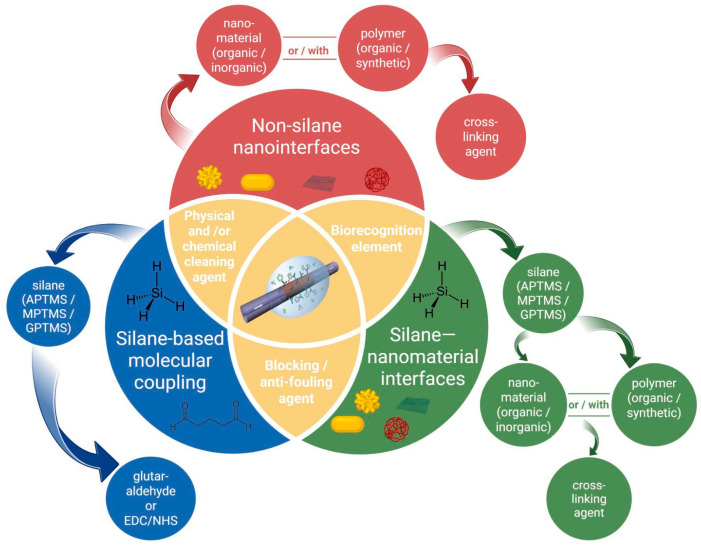
Schematic overview of surface functionalization strategies for fiber-optic biosensors used in cancer detection. The diagram is color-coded to distinguish three principal approaches: silane-based molecular coupling (blue), silane–nanomaterial interfaces (green), and non-silane nanointerfaces (red). All strategies originate from a common surface cleaning and activation step and incorporate biorecognition elements for specific target recognition. The yellow regions denote steps and components shared across all approaches, including physical and/or chemical cleaning, biorecognition elements, and the use of blocking or antifouling agents to suppress nonspecific adsorption.

Based on the functionalization concepts discussed throughout this manuscript, Figure 11 provides a consolidated schematic overview of the principal surface functionalization strategies used in fiber-optic biosensors for cancer detection.

## 4. Computational Methods in Functionalized Optical Sensing for Oncology

Because fiber-optic sensors operate through evanescent-field interactions, even small changes in the refractive index, thickness, surface charge, or dielectric properties of functional layers can dramatically alter measurable outputs such as resonance wavelength, phase, or transmission intensity. Experimental optimization of these parameters is challenging, time-consuming, and often difficult to interpret without a quantitative understanding of the underlying light-matter interaction mechanisms. Consequently, according to the latest trend, computational methods have become essential tools for predicting sensor response, guiding material and geometry selection, and rationally engineering functional layers for enhanced biomolecular recognition [143]. There are three main computational approaches used for fiber optic sensing studies: electromagnetic numerical simulation methods [110,144,145,146,147,148,149], analytical and semi-analytical optical modeling [38,106,150], hybrid physics combined with machine learning [107,109,151,152,153,154]. Each method addresses distinct aspects of light-matter interaction, sensor geometry, and performance optimization in fiber optic biosensing systems.

Electromagnetic simulation methods form the core of fiber optic sensing computational studies, enabling accurate modeling plasmonic coupling, mode propagation, confinement loss, and field localization in complex fiber and photonic structures, with FEM being the most widely used due to its geometric and material flexibility [110,144,145,146,147,148,149,155]. Analytical and semi-analytical models (TMM, Fresnel multilayer analysis, Drude-Lorentz material modeling) provide fast, physically intuitive tools for planar and layered SPR configurations, mainly for parameter sweeps, thickness optimization, and resonance interpretation [38,106,150]. Hybrid physics-machine learning approaches combine simulation-generated data with machine learning models to predict optical responses, optimize sensor parameters, and classify analytes, significantly reducing computational cost while retaining physical consistency [107,109,151,152,153,154].

For the implementation of these computational methods in fiber-optic biosensing studies, a well-defined set of material, geometrical, and analyte-related data is required. This data set is essential for modeling, because material properties determine optical dispersion and plasmonic response, geometrical parameters define mode confinement and light-matter interaction strength, and analyte-related data enable modeling of refractive-index perturbations associated with normal and cancerous states, which mainly manage resonance shifts and sensing performance [38,106,107,109,110,144,145,146,147,148,149,150,151,152,153,154,155]. Accordingly, Table 2 summarizes representative computational studies in this field by systematically categorizing the fiber sensor type, targeted cancer cell or biomarker, surface functionalization strategy, modeling and simulation methodology, key simulated performance metrics, and corresponding references.

## 5. Challenges and Future Perspectives

The performance of optical fiber biosensors depends heavily on surface functionalization, and the strategy chosen depends on the intended application. This extensive review defined the common procedures in surface modification techniques that were successful in identifying cancer biomarkers. However, there are still challenges that include stability and robustness of functional layers, biofouling and non-specific adsorption in real samples, control of bioreceptor orientation and surface architecture, reproducibility, and integration with packaging, microfluidics, and catheters.

Another persistent challenge in optical fiber biosensors is the long-term stability of the functional layers. Silane monolayers, thiol-on-gold SAMs, and polymer or nanoparticle coatings can undergo hydrolysis, oxidation, or delamination over time, especially when subjected to complex biological fluids or repeated wet and dry cycles. Ensuring robust and durable surface functionalization remains a major barrier to reliable clinical deployment.

The functionalized biosensors can be stored and utilized not over a week of detection, even in successful platforms like the silanized—CD44 ball-tip resonators and taper sensors, showing the deterioration of sensing afterwards [56,120].

Reviews of optical biosensors frequently stress that, even with the most common blockers like BSA or m-PEG amines, non-specific adsorption of serum proteins, lipids, and cells on functionalized surfaces continues to be a major source of noise and signal drift [156].

The difficulty of achieving true antifouling behavior is highlighted by the fact that fiber-optic cancer biosensors that reach ultralow limits of detection for CD44 or sHER2 frequently report performance in buffer and diluted serum with more limited results in undiluted or extremely complex matrices [57].

Antibody orientation still remains a difficult design challenge for surface functionalization, as demonstrated by recent work using Protein G on fiber surfaces for ALDH1A1 detection, which explicitly demonstrates enhanced response when antibody orientation is regulated [122]. Meanwhile, the packaging of optical fiber biosensors presents another limitation, as embedding the functionalized region into flow cells, microfluidic chips, or catheters is challenging without damaging the bioreceptor layers and not breaking the biosensor by itself. For instance, precise packaging was needed to maintain both the surface chemistry and the optical response while incorporating a CD44-functionalized ball resonator into an intrathecal catheter for attomolar detection [57,157].

Furthermore, there is a gap between functionalized-fiber prototypes and clinically validated diagnostic tests, and while many optical biosensors achieve excellent detection limits, they are usually tested on small cohorts or spiked samples, according to several recent reviews [11]. Despite extensive research dedicated to cancer biomarker detection, the vast majority of biosensing technologies remain in the laboratory prototyping stage, with commercial translation being a rare exception. However, a few pioneering platforms have successfully entered the market using distinct technological approaches. One known example is the inPROBE^®^ platform, designed for the specific in vivo detection of molecular markers like HER2 directly within patient tissue [158]. Another example is SEDI-ATI platform that have focused on structural “optical biopsy” by integrating a micro-scale optical fiber into a fine needle capable of penetrating the skin without surgical incision. Unlike biomarker-specific sensors, this device operates under ultrasound guidance to deliver laser excitation and collect cellular autofluorescence and light scattering data [159].

As has already been shown for ALDH1A1 and other protein biomarkers, future research will probably increase the use of orientation-controlling layers (Protein A/G, Protein G, Fc-binding peptides, engineered tags) and click-compatible linkers on fiber surfaces to methodically increase sensitivity and reproducibility [122].

As promising methods to alter the surface of optical fiber biosensors, future research may potentially concentrate on the synthesis of sophisticated nanomaterials such as metal–organic frameworks (MOFs) and other hybrid nanomaterials [10,160]. To ensure that biorecognition layers stay stable and accessible during flow, and mechanical stress, future surface functionalization solutions will need to be co-designed with packaging [161].

The future of optical fiber biosensors for cancer biomarkers detection should move towards the transition from fundamental research to industrial commercialization. To achieve this, research focus must shift towards the extensive clinical trials by implementing the robust packaging system ensuring the easy to use and mechanically stable sensing platforms.

## 6. Conclusions

The foundation of optical fiber biosensing for cancer detection is still surface functionalization, which controls the platforms’ long-term dependability, sensitivity, and specificity. Self-assembled monolayers, plasmonic nanoparticles, 2D materials, molecularly imprinted polymers (MIPs), hydrogels, and hybrid organic–inorganic nanostructures are just a few of the increasingly complex interfaces that the discipline has developed over the last ten years. These functional layers improve light-matter interactions, allow for precise control over surface chemistry, and produce high-density, stable anchoring sites for bioreceptors including peptides, antibodies, aptamers, and synthetic ligands.

However, long-term stability, resistance to nonspecific adsorption, repeatable production, and smooth integration with clinically relevant operations continue to be difficult to achieve. The development of sophisticated immobilization chemistries and next-generation coatings is still motivated by the requirement for exact control over bioreceptor orientation, surface density, and antifouling performance. Simultaneously, the potential of optical fibers is being expanded from single-analyte detectors to integrated diagnostic platforms capable of real-time, minimally invasive cancer monitoring through the development of lab-on-fiber concepts, multimodal nanostructured interfaces, and multiplexed sensing architectures.

Overall, the advancements outlined in this paper highlight the fact that strong surface chemistries, nanomaterial-enabled improvement, and biologically intelligent interface design will work in concert to shape the future of optical fiber cancer biosensing. Optical fiber biosensors are ready to move from lab prototypes to potent diagnostic instruments with real influence on early cancer detection, tailored treatment, and patient monitoring as functionalization techniques become more reliable, selective, and compatible with clinical settings.

## Figures and Tables

**Table 1:** Extensive overview of all OFBs used for the detection of cancer biomarkers.

#	Fiber Type	Main Mechanism	Material	Biorecognition Element	Cancer Cell Type (s)	Sensitivity	LoD	Ref.
1	TFBG + SMF	Multiresonance light scattering sensing	Halloysite nanotubes (HNTs)	Physical cell capture via HNT slit-like nanostructures (no molecular affinity receptor)	Breast cancer	Not given	10 cells·mL^−1^	[27]
2	D-shaped polymer OF	SPR	Gold nanofilm + CYTOP polymer	HER2-specific aptamer	Breast cancer	28,100 nm/RIU	~5.28 nM	[38]
3	MCF+SMF	SPR	GO/AuNPs/CuO nanoflowers	2-deoxy-D-glucose (2-DG), targeting GLUT receptors	Liver, lung, breast cancer	Not given	3, 2, 2, 2, 4, 10 cells/mL	[39]
4	SMF ball resonator	Refractometric	Gold thin film	Anti-CD44 antibody	Breast, colon, gastric, lung, ovarian, cervical cancers	95.76 dB·RIU^−1^	17 pM	[56]
5	SMF ball resonator	Refractometric	APTMS + Glutaraldehyde	Anti-CD44 antibody	Breast cancer	−85 to −120 dB·RIU^−1^	4.68 aM	[57]
6	SMF	Refractometric	APTES + glutaraldehyde	DNA probe	Not cancer cell-specific	0.175 dB·nM^−1^	1 nM	[59]
7	TFG	Refractometric	Black phosphorus (BP)	Anti-neuron-specific enolase (anti-NSE) antibodies	Lung cancer	NSE	1.0 pg/mL	[63]
8	SMF ball resonator	Refractometric	Zinc oxide	Anti-CD44 antibody	Breast cancer	−80.056 dB/RIU	0.8 fM	[64]
9	Unclad MMF	SPR	Gold thin film	HER2 antibody	Breast cancer	Not given	6.6 × 10^−7^ g/mL	[65]
10	SMF ball resonator	Refractometric	APTMS + Glutaraldehyde	Anti-CD44 antibody	Breast cancer	−92.1 dB·RIU^−1^	335 cells·mL^−1^	[69]
11	TFBG + ball resonator	Refractometric	APTMS, glutaraldehyde	Anti-HER2 antibody	Breast cancer	4034 dB/RIU	In buffer and in a 1/10 diluted serum of 151.5 ag/mL and 3.7 pg/mL, respectively	[70]
12	Microfiber	Refractometric	APTES + Glutaraldehyde	Anti-HER2 antibody	Breast cancer	1867 nm·RIU^−1^	Down to 2 ng mL^−1^ in serum	[53]
13	MMF	SPR	Gold nanodisk particles	Anti-PSA antibody	Prostate cancer	5700 RIU^−1^	1.3 pg·mL^−1^	[73]
14	mTLPG	SPR	Gold nanorods	Anti-AFP antibodies	Liver cancer	3 pm/(ng/mL)	17.6 ng/mL	[74]
15	MMF+SMF	SPR	Gold thin film	Anti-EpCAM antibody	Breast cancer	1933.4 nm·RIU^−1^	1.4 cells·µL^−1^	[78]
16	U-bent MMF	SPR	Gold nanoparticles	Anti-nucleolin DNA aptamer	Retinoblastoma, meningioma, breast, cervical, and colon cancer	Not given	500 cells·mL^−1^ (bench); 50 cells·mL^−1^ (portable μSens system)	[82]
17	Multimode U-bend fiber	SPR	Gold nanoparticles	AFP antibody	Hepatocellular carcinoma	Not given	0.85 and 3.3 ng·mL^−1^ for PBS nad human serum, respectively	[83]
18	MMF	SPR	Bottom-up gold nanospheres	Anti-CA19-9 antibody	Pancreatic cancer	Not given	0.25 U/mL	[84]
19	TFBG	SPR	Gold thin film	GRP-30 antibody	Breast cancer	Not given	Single-cell level	[85]
20	D-shaped fiber	SPR	Gold thin film	Anti-hCG antibody	Prostate cancer	0.2171 nm/(µg/mL)	Not given	[86]
21	Hetero-core fiber	SPR	Gold thin film	Anti-HER2 antibody	Breast cancer	0.20666 nm/(μg/mL)	Not given	[87]
22	MMF	SPR	Gold thin film	Capture antibodies: Anti-HER, anti-EpCAM; Detection antibodies: biotinylated anti-CD9/CD63/CD81	Breast cancer	Not given	2.1 × 10^7^ particles/mL (SK-BR-3 EVs, buffer); 7 × 10^8^ particles/mL (SK-BR-3 EVs, plasma); 1.1 × 10^8^ particles/mL (MCF7 EVs, plasma)	[88]
23	Plastic-clad quartz optical fiber (HCPCF)	SPR	Au thin film	Anti-NMP22 antibody	Bladder cancer	0.092 nm·(ng mL^−1^)^−1^	0.092 ng mL^−1^	[89]
24	SMF ball resonator	Refractometric	Gold thin film	Anti-CD44 antibody	Breast cancer	1594 RIU^−1^	19.7 pM	[90]
25	SMF + TFBG	SPR	Au thin film	Anti-HER2 aptamer	Breast cancer	~0.8 nm·(µg mL^−1^)^−1^ (from ~800 pm shift at 10^−6^ g mL^−1^)	10^−12^ g mL^−1^ (8.36 fM)	[91]
26	SMF ball resonator	Refractometric	Green-synthesized gold nanoparticles	Anti-CD44 antibody	Breast cancer	1.52 dB per 10× concentration increase	0.111 pM	[92]
27	Microfiber	SPR	Gold nanorods + Black Phosphorus	HER2 antibody	Breast cancer	0.66 aM in buffer solution and 0.77 aM in 10% serum	Not given	[95]
28	D-shaped HCPCF	SPR	AuNP@GO	Anti-ProGRP antibody	Lung cancer	583,000 nm/RIU	17.1 ag/mL	[98]
29	LPG	Refractometric	Graphene oxide	Not applied	Breast cancer	Not given	270 cells/mL	[99]
30	Microfiber	Refractometric	Graphene oxide	Anti-AFP antibody	Hepatocellular carcinoma	1.11582 nm/lg (mol/L)	78 zg/mL	[100]
31	Microfiber	SPR	Ti_3_C_2_ MXene/AuNR hybrid	CAIX-specific DNA aptamer	Renal cancer	Not given	13.8 zM in pure buffer solution and 0.19 aM in 30% serum solution for CAIXproteins, 180 cells/mL for living cancer cells	[108]
32	D-shaped fiber	SPR	Au + L-cys/MoS_2_	Anti-Ferritin antibody; Anti-IgG antibody	Not cancer-cell specific	0.04 nm per µg/mL and 0.024 for IgG and ferritin, respectively	12 ng/mL and 7.2 µg/mL for ferritin and IgG, respectively	[111]
33	FBG fused to MMF	SPR	Au + PAA/CS multilayernanofilm	Probe DNA	Lung cancer	0.04 nm/nM	13.5 nM	[113]
34	Ω-shaped fiber	SPR	AuNPs/AuNRs@PDA	MUC1 DNA aptamer	Breast cancer	37.59 a.u/RIU	Not given	[114]
35	Microfiber	Refractometric	PDDA/PAA polyelectrolyte multilayer	Anti-CEA antibody	Colorectal, breast, and lung cancers	Up to ~10–12 nm·(pg mL^−1^)^−1^ in the ultralow range (200 fg mL^−1^–1 pg mL^−1^)	34.6 fg mL^−1^ (0.475 fM)	[115]
36	TFBG	SPR	PAN + Gold	Anti-CEA antibody	Pancreatic, gastric, breast, and lung cancers	0.46 dB/(µg/mL)	505.4 ng/mL	[116]
37	Tapered microfiber	Refractometric	PS@Au nanospheres	Anti-CEACAM5 antibody	Gastrointestinal, colorectal, and pancreatic, lung cancers	Not given	3.54 × 10^−17^ M and 5.27 × 10^−16^ M for pure and serum solutions, respectively	[117]
38	Peanut structure cascaded lasso shaped fiber	Refractometric	Fe_3_O_4_ microspheres	Anti-CEACAM5 antibody	Gastrointestinal, colorectal, and pancreatic, lung cancers	1747 nm/RIU	0.11 ng/mL in buffer solution	[118]
39	U-shaped thin-core fiber	Refractometric	APTES	Biotin	Lung and breast cancer	18,390 nm·RIU^−1^	49 cells·mL^−1^	[119]
40	Shallow-tapered SMF	Refractometric	APTMS + Glutaraldehyde	Anti-CD44 antibody	Breast cancer	Up to 1.33 nm/RIU	16.4 pM	[120]
41	SMF ball resonator	Refractometric	APTMS + Glutaraldehyde	Anti-CD44 antibody	Breast cancer	−85 dB/RIU (in the dynamic conditions)	Femtomolar range	[121]
42	SMF + EBF (Enhanced Backscattering Fiber)	Refractometric	APTMS + Glutaraldehyde	Anti-ALDH1A1	Breast, lung, colorectal, prostate cancers and lymphoma	Up to 92.4 dB·RIU^−1^	172 fM	[122]

**Table 2:** Computational studies on surface functionalization of fiber optic biosensors for oncological detection.

#	Fiber Sensor Type	Cancer Cell Type	Surface Functionalization	Modeling/Simulation Method	Simulated Outputs	Ref.
1	D-shaped polymer optical fiber (POF) SPR biosensor	HER2 protein	Gold + HER2 aptamer	FEM + multilayer Transfer Matrix Method (TMM)	Transmission spectra shifts for gold thicknesses, resonant wavelength redshift as gold thickness increases, sensitivity, FWHM (Full Width at Half Maximum) variations for each thickness, Peak FOM	[38]
2	PCF-SPR sensor	Basal, HeLa, PC-12, MDA-MB-231, MCF-7	Hybrid Au/Ti_3_C_2_T_x_ (MXene) thin-film coating	FEM + Machine Learning	Resonance wavelength shift, wavelength sensitivity, confinement loss, effective refractive index (Neff), resolution, FOM, ML-predicted sensitivity and Neff	[109]
3	PCF-SPR biosensor	Basal skin, HeLa, Jurkat, PC-12, MDA-MB-231, MCF-7	Au + MXene	FEM (Finite Element Method) with PML	Effective index of core & SPP mode, Confinement loss, Mode field distributions, Resonance wavelength (RW) shift, Sensitivity, FOM (Figure of Merit), Resolution	[110]
4	Single-core PCF-based SPR biosensor with U-shaped analyte channel	Basal (skin), Jurkat (blood), PC12/adrenocortical (adrenal gland) cancer cells	Gold (Au) plasmonic layer with V_2_O_5_ adhesion nanolayer	FEM	Resonance wavelength shift, confinement loss, wavelength sensitivity, resolution, FOM	[144]
5	Terahertz porous-core MSF (microstructure fiber) biosensor	Breast, skin, gastric cancer cells	Geometric functionalization (porous-core structure, Zeonex polymer matrix)	Full-vector FEM (Finite Element Method) with PML (Perfectly Matched Layer) boundary conditions	Relative sensitivity, Effective Material Loss, Confinement Loss, Numerical Aperture, Effective Mode Area, mode-field distribution	[155]
6	PCF (Photonic crystal fiber) SPR (Surface Plasmon Resonance) (PCF-SPR) biosensor	MDAMB-231, MCF-7, PC12, HeLa, Jurkat cells	TiO_2_ adhesion layer + Au plasmonic thin film	Full-vector FEM with PML	Confinement loss, resonance wavelength shift, effective index matching, amplitude sensitivity, refractive index resolution	[145]
7	Lasso-shaped SMF (single mode) fiber-laser biosensor	CEACAM5 proteins	Silanization + Anti-CEACAM5 antibody	Full-vector BPM (Beam Propagation Method) with PML conditions	Optical field distribution in straight vs. bent single-mode fiber (SMF), mode coupling between core and cladding, formation of cladding modes and Multimode Interference (MMI), evanescent field depth, sensitivity	[146]
8	Hybrid plasmonic-photonic crystal (MIM + 1D PC PBG) biosensor	Basal cell carcinoma	Ag/GaAs/air (no bioreceptors)	FDTD (Finite-Difference Time-Domain) simulation; TLM validation	Transmission spectrum, PBG wavelength shift, sensitivity, FOM	[147]
9	PCF-SPR biosensor	Basal skin, HeLa, Jurkat, PC-12, MDA-MB-231, MCF-7	Gold + TiO_2_	FEM (Finite Element Method) with PML	Confinement loss, Mode field distributions, Resonance wavelength (RW) shift, Sensitivity, FOM, Resolution	[148]
10	PCF-SPR biosensor	Basal, HeLa, Jurkat, PC-12, MDA-MB-231, MCF-7	Gold nanowire	FEM (Finite Element Method), Drude-Lorentz model	CL spectra, resonance wavelength, WS (wavelength sensitivity), AS (amplitude sensitivity), resolution	[149]
11	LSPR (Localized Surface Plasmon Resonance) biosensor	BRCA-1 and BRCA-2 genetic 12breast cancer cells	Au film + graphene layers + immobilized probe DNA	Analytical multilayer Fresnel transfer matrix modeling; SPR angle & SRF simulations	SPR angle, Surface Resonance Frequency shift reflectance curves, effect of graphene layer number	[150]
12	SPR biosensor	Jurkat, HeLa, PC12, MDA-MB-231, MCF7 cells	Perfluorinated polymer + Ag/MoS_2_/polymer/graphene	TMM, Multilayer optical model	SPR angle, power loss spectrum, FWHM, sensitivity, FOM, LoD	[106]
13	PCF-SPR biosensor	MCF-7, MDA-MB-231 cells	Au + TiO_2_	FEM with PML & Machine Learning analysis	Neff(core), Neff (SPP), confinement loss, sensitivity, resonance wavelength shift	[151]
14	Circular shaped HCF—SPR biosensor	A549, HepG2, MCF-7, basal cells	Graphene + MoS_2_ + Gold	FEM (Finite Element Method) + Machine Leaning	SPR wavelength shift, Confinement loss, wavelength sensitivity, ML-predicted optics	[107]
15	PCF-SPR biosensor	Basal cancer, MDA-MB-231, MCF-7, Jurkat, PC12, HeLa cells	Gold (Au)	FEM (Finite Element Method) + Machine Learning	Resonance wavelength shift; confinement loss (dB/cm); effective refractive index (Neff); wavelength sensitivity	[152]
16	Kretschmann-configuration plasmonic biosensor (prism-coupled SPR)	Brain tumor biomarkers	Graphene/Ag/WS_2_ multilayer	FEM (Finite Element Method) + transfer matrix modeling + Machine Learning	Angular reflectance spectra, resonance angle shift, sensitivity, detection limit, figure of merit, ML-simulation correlation	[153]
17	Open D-channel PCF-SPR sensor	Basal, Jurkat, HeLa, PC12, MDA-MB-231, MCF7 cells	Au/TiO_2_ thin-film bilayer	FEM with PML	Resonance wavelength shift; wavelength sensitivity; amplitude sensitivity; resolution; FOM	[154]

## Data Availability

All data are available and provided.

## References

[1] World Health Organization Cancer. https://www.who.int/news-room/fact-sheets/detail/cancer.

[2] Crosby D., Bhatia S., Brindle K.M., Coussens L.M., Dive C., Emberton M., Esener S., Fitzgerald R.C., Gambhir S.S., Kuhn P. (2022). Early detection of cancer. Science.

[3] Soares M.S., Vidal M., Santos N.F., Costa F.M., Marques C., Pereira S.O., Leitão C. (2021). Immunosensing based on optical fiber technology: Recent advances. Biosensors.

[4] Fitzgerald R.C., Antoniou A.C., Fruk L., Rosenfeld N. (2022). The future of early cancer detection. Nat. Med..

[5] Leitão C., Pereira S.O., Marques C., Cennamo N., Zeni L., Shaimerdenova M., Ayupova T., Tosi D. (2022). Cost-Effective Fiber Optic Solutions for Biosensing. Biosensors.

[6] Zubair H., Begum M., Moradi F., Rahman A.K.M.M., Mahdiraji G.A., Oresegun A., Louay G.T., Omar N.Y., Khandaker M.U., Adikan F.R.M. (2020). Recent Advances in Silica Glass Optical Fiber for Dosimetry Applications. IEEE Photonics J..

[7] Zubia J., Arrue J. (2001). Plastic Optical Fibers: An Introduction to Their Technological Processes and Applications. Opt. Fiber Technol..

[8] Peters K. (2010). Polymer optical fiber sensors—A review. Smart Mater. Struct..

[9] Wandermur G., Rodrigues D., Allil R., Queiroz V., Peixoto R., Werneck M., Miguel M. (2014). Plastic optical fiber-based biosensor platform for rapid cell detection. Biosens. Bioelectron..

[10] Kaur B., Kumar S., Kaushik B.K. (2022). Recent advancements in optical biosensors for cancer detection. Biosens. Bioelectron..

[11] Azab M.Y., Hameed M.F.O., Obayya S.S.A. (2023). Overview of Optical Biosensors for Early Cancer Detection: Fundamentals, Applications and Future Perspectives. Biology.

[12] Wallace G.Q., Masson J.-F. (2020). From single cells to complex tissues in applications of surface-enhanced Raman scattering. Analyst.

[13] Lin C., Li Y., Peng Y., Zhao S., Xu M., Zhang L., Huang Z., Shi J., Yang Y. (2023). Recent development of surface-enhanced Raman scattering for biosensing. J. Nanobiotechnol..

[14] (2018). Home. Nanobiosensors and Fluorescence Based Biosensors: An Overview. https://www.SID.ir.

[15] Fan X., White I.M., Shopova S.I., Zhu H., Suter J.D., Sun Y. (2008). Sensitive optical biosensors for unlabeled targets: A review. Anal. Chim. Acta.

[16] Jin F., Xu Z., Cao D., Ran Y., Guan B.-O. (2024). Fiber-Optic Biosensors for Cancer Theranostics: From in Vitro to in Vivo. Photonic Sens..

[17] Bureau B., Zhang X.H., Smektala F., Adam J.-L., Troles J., Ma H.-L., Boussard-Plèdel C., Lucas J., Lucas P., Le Coq D. (2004). Recent advances in chalcogenide glasses. J. Non-Cryst. Solids.

[18] Bureau B., Boussard C., Cui S., Chahal R., Anne M.L., Nazabal V., Sire O., Loréal O., Lucas P., Monbet V. (2014). Chalcogenide optical fibers for mid-infrared sensing. Opt. Eng..

[19] Lucas P., Riley M.R., Boussard-Plédel C., Bureau B. (2006). Advances in chalcogenide fiber evanescent wave biochemical sensing. Anal. Biochem..

[20] Sanghera J.S., Shaw L., Aggarwal I.D. (2002). Applications of chalcogenide glass optical fibers. C. R. Chim..

[21] Lucas P., Coleman G.J., Jiang S., Luo T., Yang Z. (2015). Chalcogenide glass fibers: Optical window tailoring and suitability for bio-chemical sensing. Opt. Mater..

[22] Li X., Chen N., Zhou X., Zhang Y., Zhao Y., Nguyen L.V., Ebendorff-Heidepriem H., Warren-Smith S.C. (2022). In-situ DNA detection with an interferometric-type optical sensor based on tapered exposed core microstructured optical fiber. Sens. Actuators B Chem..

[23] Barozzi M., Manicardi A., Vannucci A., Candiani A., Sozzi M., Konstantaki M., Pissadakis S., Corradini R., Selleri S., Cucinotta A. (2016). Optical Fiber Sensors for Label-Free DNA Detection. J. Light. Technol..

[24] Lyu S., Wu Z., Shi X., Wu Q. (2022). Optical Fiber Biosensors for Protein Detection: A Review. Photonics.

[25] Lukose J., Chidangil S., George S.D. (2021). Optical technologies for the detection of viruses like COVID-19: Progress and prospects. Biosens. Bioelectron..

[26] Vajhadin F., Mazloum-Ardakani M., Sanati A., Haghniaz R., Travas-Sejdic J. (2022). Optical cytosensors for the detection of circulating tumour cells. J. Mater. Chem. B.

[27] Zu L., Chen Y., Xie J., Liu W., Feng Y., Zhang Z., Zhao X., Ma Y., Fang Q., Li K. (2023). In situ tumor cells detection using nanotube-functionalized & microfluidic-controlling multiresonance optical fiber. Sens. Actuators B Chem..

[28] Soler M., Lechuga L.M. (2021). Biochemistry strategies for label-free optical sensor biofunctionalization: Advances towards real applicability. Anal. Bioanal. Chem..

[29] Lin P.-H., Li B.-R. (2019). Antifouling strategies in advanced electrochemical sensors and biosensors. Analyst.

[30] Ramola A., Shakya A.K., Bergman A. (2025). Comprehensive Analysis of Advancement in Optical Biosensing Techniques for Early Detection of Cancerous Cells. Biosensors.

[31] Dabagh S., Singh R., Borri C., Chiavaioli F. (2025). Functional Nanomaterial Coatings on Optical Fibers: Toward Enhanced Biosensing Performance. IEEE Sens. Rev..

[32] Szunerits S., Spadavecchia J., Boukherroub R. (2014). Surface plasmon resonance: Signal amplification using colloidal gold nanoparticles for enhanced sensitivity. Rev. Anal. Chem..

[33] Topor C.-V., Puiu M., Bala C. (2023). Strategies for Surface Design in Surface Plasmon Resonance (SPR) Sensing. Biosensors.

[34] Gade A., Sharma A., Srivastava N., Flora S. (2022). Surface plasmon resonance: A promising approach for label-free early cancer diagnosis. Clin. Chim. Acta.

[35] Lobry M., Loyez M., Hassan E.M., Chah K., DeRosa M.C., Goormaghtigh E., Wattiez R., Caucheteur C. (2020). Multimodal plasmonic optical fiber grating aptasensor. Opt. Express.

[36] Leung A., Shankar P.M., Mutharasan R. (2007). A review of fiber-optic biosensors. Sens. Actuators B Chem..

[37] Wu Q., Qu Y., Liu J., Yuan J., Wan S.-P., Wu T., He X.-D., Liub B., Liuc D., Ma Y. (2020). Singlemode-Multimode-Singlemode Fiber Structures for Sensing Applications—A Review. IEEE Sens. J..

[38] Wu X., Wang Y., Zhang J., Zhang Y., Rao X., Chen C., Liu H., Deng Y., Liao C., Smietana M.J. (2023). A D-Shaped Polymer Optical Fiber Surface Plasmon Resonance Biosensor for Breast Cancer Detection Applications. Biosensors.

[39] Singh R., Kumar S., Liu F.-Z., Shuang C., Zhang B., Jha R., Kaushik B.K. (2020). Etched multicore fiber sensor using copper oxide and gold nanoparticles decorated graphene oxide structure for cancer cells detection. Biosens. Bioelectron..

[40] Guo T., González-Vila Á., Loyez M., Caucheteur C. (2017). Plasmonic optical fiber-grating immunosensing: A review. Sensors.

[41] Zhong X., Xie Q., Liu Y., He Y., Zhong N., Zhang Z., Karimi-Maleh H., Peng X., Lichtfouse E. (2024). Recent advances in optical fiber grating sensors for detection of organic substances. Chem. Eng. J..

[42] Lee B.H., Kim Y.H., Park K.S., Eom J.B., Kim M.J., Rho B.S., Choi H.Y. (2012). Interferometric fiber optic sensors. Sensors.

[43] Elsherif M., Salih A.E., Muñoz M.G., Alam F., AlQattan B., Antonysamy D.S., Zaki M.F., Yetisen A.K., Park S., Wilkinson T.D. (2022). Optical Fiber Sensors: Working Principle, Applications, and Limitations. Adv. Photonics Res..

[44] Shadab A., Raghuwanshi S.K., Kumar S. (2022). Advances in Micro-Fabricated Fiber Bragg Grating for Detection of Physical, Chemical, and Biological Parameters—A Review. IEEE Sens. J..

[45] Chiavaioli F., Baldini F., Tombelli S., Trono C., Giannetti A. (2017). Biosensing with optical fiber gratings. Nanophotonics.

[46] Sun D., Ran Y., Wang G. (2017). Label-free detection of cancer biomarkers using an in-line taper fiber-optic interferometer and a fiber bragg grating. Sensors.

[47] Quero G., Consales M., Severino R., Vaiano P., Boniello A., Sandomenico A., Ruvo M., Borriello A., Diodato L., Zuppolini S. (2016). Long period fiber grating nano-optrode for cancer biomarker detection. Biosens. Bioelectron..

[48] Loyez M., Lobry M., Wattiez R., Caucheteur C. (2019). Optical fiber gratings immunoassays. Sensors.

[49] Chiang C.-C., Yeh Y.-T., Wang T.-E., Hsu H.-C., Wen H.-Y. (2024). A pathway for detection of gastric cancer biomarkers via using a layer-by-layer coated D-shaped grinding long-period fiber grating sensor. Anal. Chim. Acta.

[50] Homola J. (2008). Surface Plasmon Resonance Sensors for Detection of Chemical and Biological Species. Chem. Rev..

[51] Bekmurzayeva A., Nurlankyzy M., Abdossova A., Myrkhiyeva Z., Tosi D. (2024). All-fiber label-free optical fiber biosensors: From modern technologies to current applications [Invited]. Biomed. Opt. Express.

[52] Zhou W., Wei Y., Wang Y., Li K., Yu H., Wu Y. (2021). Ultrasensitive interferometers based on zigzag-shaped tapered optical microfibers operating at the dispersion turning point. Opt. Express.

[53] Sun D., Fu Y., Yang Y. (2020). Label-free detection of breast cancer biomarker using silica microfiber interferometry. Opt. Commun..

[54] Lobry M., Fasseaux H., Loyez M., Chah K., Goormaghtigh E., Wattiez R., Chiavaioli F., Caucheteur C. (2021). Plasmonic Fiber Grating Biosensors Demodulated through Spectral Envelopes Intersection. J. Light. Technol..

[55] Rivero P.J., Goicoechea J., Arregui F.J. (2019). Layer-by-Layer Nano-assembly: A Powerful Tool for Optical Fiber Sensing Applications. Sensors.

[56] Bekmurzayeva A., Ashikbayeva Z., Myrkhiyeva Z., Nugmanova A., Shaimerdenova M., Ayupova T., Tosi D. (2021). Label-free fiber-optic spherical tip biosensor to enable picomolar-level detection of CD44 protein. Sci. Rep..

[57] Bekmurzayeva A., Ashikbayeva Z., Assylbekova N., Myrkhiyeva Z., Dauletova A., Ayupova T., Shaimerdenova M., Tosi D. (2022). Ultra-wide, attomolar-level limit detection of CD44 biomarker with a silanized optical fiber biosensor. Biosens. Bioelectron..

[58] Nedoma J., Krizan D., Stipal J., Pereira L., Bekmurzayeva A., Tosi D., Costa M.B., Leal-Junior A., Martinek R., Marques C. (2025). Decade of advancements in light–matter interaction-based optical fiber biosensing: Innovations, challenges, and future directions. Adv. Photonics.

[59] Shen C., Chen X., Huang Z., Wang Z., Liu J., Deng H., Liu D., Shu F. (2022). High sensitivity and fast response optical fiber nucleic acid sensor. Opt. Laser Technol..

[60] Spence J. (2022). Chemical Safety-Special Hazards Safe Work Procedure (SWP-009), Piranha Solution, Occupational Health, Safety and Environment.

[61] Schmidt H.G. (2021). Safe Piranhas: A Review of Methods and Protocols. ACS Chem. Health Saf..

[62] Kumar S., Kaushik B.K., Singh R., Chen N.-K., Yang Q.S., Zhang X., Wang W., Zhang B. (2019). LSPR-based cholesterol biosensor using a tapered optical fiber structure. Biomed. Opt. Express.

[63] Zhou L., Liu C., Sun Z., Mao H., Zhang L., Yu X., Zhao J., Chen X. (2019). Black phosphorus based fiber optic biosensor for ultrasensitive cancer diagnosis. Biosens. Bioelectron..

[64] Paltusheva Z.U., Ashikbayeva Z., Tosi D., Gritsenko L.V. (2022). Highly Sensitive Zinc Oxide Fiber-Optic Biosensor for the Detection of CD44 Protein. Biosensors.

[65] Loyez M., Lobry M., Hassan E.M., DeRosa M.C., Caucheteur C., Wattiez R. (2021). HER2 breast cancer biomarker detection using a sandwich optical fiber assay. Talanta.

[66] Rostami S., Zibaii M.I., Babakhani-Fard M.-M., Layeghi A., Latifi H. (2025). Sensitivity enhancement and thermal compensation of LSPR-based optical fibre refractive index sensor using annealing of Au nanoparticles. Sens. Actuators A Phys..

[67] Verding P., Joy R.M., Reenaers D., Kumar R.S.N., Rouzbahani R., Jeunen E., Thomas S., Desta D., Boyen H.-G., Pobedinskas P. (2023). The Influence of UV–Ozone, O_2_ Plasma, and CF_4_ Plasma Treatment on the Droplet-Based Deposition of Diamond Nanoparticles. ACS Appl. Mater. Interfaces.

[68] Meng X., O’Hare D., Ladame S. (2023). Surface immobilization strategies for the development of electrochemical nucleic acid sensors. Biosens. Bioelectron..

[69] Nurlankyzy M., Kantoreyeva K., Myrkhiyeva Z., Ashikbayeva Z., Baiken Y., Kanayeva D., Tosi D., Bekmurzayeva A. (2024). Label-free optical fiber biosensor for the detection of CD44-expressing breast cancer cells. Sens. BioSens. Res..

[70] Sypabekova M., Amantayeva A., Vangelista L., González-Vila Á., Caucheteur C., Tosi D. (2022). Ultralow Limit Detection of Soluble HER2 Biomarker in Serum with a Fiber-Optic Ball-Tip Resonator Assisted by a Tilted FBG. ACS Meas. Sci. Au.

[71] Murugan D., Tintelott M., Amiri H., Kasavetov M., Besedin D., Ingebrandt S., Vu X.T., Pachauri V. (2023). Comparative Study of Surface Activation Steps for Thermally Grown Oxide Interface and Optimal Silanization. Phys. Status Solidi (A).

[72] Arghir I., Spasic D., Verlinden B.E., Delport F., Lammertyn J. (2015). Improved surface plasmon resonance biosensing using silanized optical fibers. Sens. Actuators B Chem..

[73] Kim H.-M., Uh M., Jeong D.H., Lee H.-Y., Park J.-H., Lee S.-K. (2019). Localized surface plasmon resonance biosensor using nanopatterned gold particles on the surface of an optical fiber. Sens. Actuators B Chem..

[74] Li X., Wang Z., Hu L., Xia J., Li S., Kuai Y., Fu W., Cao Z., Wang M., Yu B. (2025). Rapid and Sensitive Detection of Liver Cancer Markers Based on a Microtapered Long-Period Grating Sensor Coated with Gold Nanorods. IEEE Sens. J..

[75] Boccafoschi F., Fusaro L., Cannas M. (2018). Immobilization of peptides on cardiovascular stent. Functionalised Cardiovascular Stents.

[76] Qu W., Chen Y., Liu S., Luo L. (2025). Advances and Prospects of Nanomaterial Coatings in Optical Fiber Sensors. Coatings.

[77] Singh M., Kaur N., Comini E. (2020). The role of self-assembled monolayers in electronic devices. J. Mater. Chem. C.

[78] Zhu S., Xie Z., Chen Y., Liu S., Kwan Y.-W., Zeng S., Yuan W., Ho H.-P. (2022). Real-Time Detection of Circulating Tumor Cells in Bloodstream Using Plasmonic Fiber Sensors. Biosensors.

[79] Mekuye B., Abera B. (2023). Nanomaterials: An overview of synthesis, classification, characterization, and applications. Nano Sel..

[80] Adul-Rasool A.A., Athair D.M., Zaidan H.K., Rheima A.M., Al-Sharify Z.T., Mohammed S.H., Kianfar E. (2024). 0,1,2,3D nanostructures, types of bulk nanostructured materials, and drug nanocrystals: An overview. Cancer Treat. Res. Commun..

[81] Baig N., Kammakakam I., Falath W. (2021). Nanomaterials: A review of synthesis methods, properties, recent progress, and challenges. Mater. Adv..

[82] Gupta R., Prakash N., Paul D., Mukherji S. (2023). Anti-nucleolin aptamer mediated specific detection of cancer cells by Localized Surface Plasmon Resonance-based U-bent optical fiber. Biosens. Bioelectron. X.

[83] Liang G., Zhao Z., Wei Y., Liu K., Hou W., Duan Y. (2015). Plasma enhanced label-free immunoassay for alpha-fetoprotein based on a U-bend fiber-optic LSPR biosensor. RSC Adv..

[84] Kim H.-M., Yang S.-C., Park J.-H., Lee S.-K. (2024). Fabrication of Top–Down-Based Optical Fiber Nanoprobes and Their Diagnostic Application for Pancreatic Cancer. IEEE Sens. J..

[85] Chen X., Xu P., Lin W., Jiang J., Qu H., Hu X., Sun J., Cui Y. (2022). Label-free detection of breast cancer cells using a functionalized tilted fiber grating. Biomed. Opt. Express.

[86] Zamri A., Mustafa M., Awang N., Zalkepali N., Mahmud N., Muhammad N. (2024). Fiber-laser based on D-shaped fiber biosensor for prostate cancer biomarker detection. Mater. Today Proc..

[87] Liang M., Li X., Chen Y. SPR-based fiber optic biosensor for the detection of HER2. Proceedings of the 2024 International Conference on Optoelectronic Information and Optical Engineering (OIOE 2024).

[88] Yildizhan Y., Driessens K., Tsao H.S.K., Boiy R., Thomas D., Geukens N., Hendrix A., Lammertyn J., Spasic D. (2023). Detection of Breast Cancer-Specific Extracellular Vesicles with Fiber-Optic SPR Biosensor. Int. J. Mol. Sci..

[89] Wang S., Cheng K., Chen S., Wei S., Ding L., Yang L., Che T., Wang N. (2025). Early Detection and Screening of Bladder Cancer Based on a Novel Optical Fiber Surface Plasmon Resonance Sensor. IEEE Sens. J..

[90] Tosi D., Ashikbayeva Z., Bekmurzayeva A., Myrkhiyeva Z., Rakhimbekova A., Ayupova T., Shaimerdenova M. (2021). Optical fiber ball resonator sensor spectral interrogation through undersampled klt: Application to refractive index sensing and cancer biomarker biosensing. Sensors.

[91] Lobry M., Loyez M., Debliquy M., Chah K., Goormaghtigh E., Caucheteur C. (2022). Electro-plasmonic-assisted biosensing of proteins and cells at the surface of optical fiber. Biosens. Bioelectron..

[92] Ashikbayeva Z., Bekmurzayeva A., Myrkhiyeva Z., Assylbekova N., Atabaev T.S., Tosi D. (2023). Green-synthesized gold nanoparticle-based optical fiber ball resonator biosensor for cancer biomarker detection. Opt. Laser Technol..

[93] Li J., Wang H., Li Z., Su Z., Zhu Y. (2020). Preparation and application of metal nanoparticals elaborated fiber sensors. Sensors.

[94] Song S., Kim K.Y., Lee S.H., Kim K.K., Lee K., Lee W., Jeon H., Ko S.H. (2021). Recent Advances in 1D Nanomaterial-Based Bioelectronics for Healthcare Applications. Adv. NanoBiomed Res..

[95] Liang H., Zhou L., Chen P., Zheng J., Huang Y., Liang J., Zhong J., Huang Y., Yu M., Guan B.-O. (2022). Optical Microfiber with a Gold Nanorods–Black Phosphorous Nanointerface: An Ultrasensitive Biosensor and Nanotherapy Platform. Anal. Chem..

[96] Lutomia D., Poria R., Kala D., Garg P., Nagraik R., Kaushal A., Gupta S., Kumar D. (2025). 2D nanomaterials in biosensing: Synthesis, characterization, integration in biosensors and their applications. Biosens. Bioelectron. X.

[97] Yang W., Jiang M., Jiang S., Du L., Cheng Y., Li P., Wang C. (2022). Design and fabrication of Gr/Ag-coated tilted grating sensor for ultra-sensitive detection of DNA hybridization. Sens. Actuators B Chem..

[98] Qiu H., Yao Y., Dong Y., Tian J. (2024). Fiber-optic immunosensor based on a Fabry–Perot interferometer for single-molecule detection of biomarkers. Biosens. Bioelectron..

[99] Sun J., Jiang H., Chavan K.J., Coutts A.S., Chen X. (2025). Graphene Oxide-Functionalized Optical Sensor for Label-Free Detection of Breast Cancer Cells. ACS Appl. Nano Mater..

[100] Deng X., Peng Y., Long R. (2025). Microfiber biosensor for detection of ultra-low concentration alpha-fetoprotein based on graphene oxide. Opt. Fiber Technol..

[101] Zhang Y., Zhou L., Qiao D., Liu M., Yang H., Meng C., Miao T., Xue J., Yao Y. (2022). Progress on Optical Fiber Biochemical Sensors Based on Graphene. Micromachines.

[102] Lee J., Kim J., Kim S., Min D.-H. (2016). Biosensors based on graphene oxide and its biomedical application. Adv. Drug Deliv. Rev..

[103] Qin Z., Zhang J., Li S. (2023). Molybdenum Disulfide as Tunable Electrochemical and Optical Biosensing Platforms for Cancer Biomarker Detection: A Review. Biosensors.

[104] Ye L., Gan X., Schirhagl R. (2024). Two-Dimensional MoS_2_-Based Photodetectors. Sustainability.

[105] Sinha A., Dhanjai, Tan B., Huang Y., Zhao H., Dang X., Chen J., Jain R. (2018). MoS2 nanostructures for electrochemical sensing of multidisciplinary targets: A review. TrAC Trends Anal. Chem..

[106] Kaur B., Kumar S., Kaushik B.K. (2021). 2D Materials-Based Fiber Optic SPR Biosensor for Cancer Detection at 1550 nm. IEEE Sens. J..

[107] Huraiya A., Shoshi M.S., Chakrabarti K., Tabata H., Ramaraj S.G., Razzak S.M.A., Eid M.M.A., Rashed A.N.Z. (2025). Ultra-Sensitive Au-based Circular Photonic Fibers Based Surface Plasmonic Resonance Biosensor for Various Cancer Level Diagnostics and Detection. Plasmonics.

[108] Li H., Huang T., Yuan H., Lu L., Cao Z., Zhang L., Yang Y., Yu B., Wang H. (2023). Combined Ultrasensitive Detection of Renal Cancer Proteins and Cells Using an Optical Microfiber Functionalized with Ti_3_C_2_ MXene and Gold Nanorod-Nanosensitized Interfaces. Anal. Chem..

[109] Kumar A., Verma P., Jindal P. (2022). Surface plasmon resonance sensor based on MXene coated PCF for detecting the cancer cells with machine learning approach. Microelectron. Eng..

[110] Lu M., He Y., Xi S., Zhong P., Zhang Y., Tian H., Wang Y., Lu H., Hu J., Tang J. (2025). High-Sensitivity MXene-Functionalized Photonic Crystal Fiber Surface Plasmon Resonance Sensor with Dual Rectangular Grooves for Cancer Detection. Sensors.

[111] Thawany P., Khanna A., Tiwari U.K., Deep A. (2023). L-cysteine/MoS2 modified robust surface plasmon resonance optical fiber sensor for sensing of Ferritin and IgG. Sci. Rep..

[112] Cennamo N., Pasquardini L., Arcadio F., Vanzetti L.E., Bossi A.M., Zeni L. (2019). D-shaped plastic optical fibre aptasensor for fast thrombin detection in nanomolar range. Sci. Rep..

[113] Li X., Gong P., Zhao Q., Zhou X., Zhang Y., Zhao Y. (2022). Plug-in optical fiber SPR biosensor for lung cancer gene detection with temperature and pH compensation. Sens. Actuators B Chem..

[114] Kong X., Li M., Xiao W., Li Y., Luo Z., Shen J.-W., Duan Y. (2024). Ω-Shaped fiber optic LSPR coated with hybridized nanolayers for tumor cell sensing and photothermal treatment. Talanta.

[115] Wei Y., Zhou W., Wu Y., Zhu H. (2021). High Sensitivity Label-Free Quantitative Method for Detecting Tumor Biomarkers in Human Serum by Optical Microfiber Couplers. ACS Sens..

[116] Li J., Liu X., Sun H., Xi J., Chang C., Deng L., Yang Y., Li X. (2024). Optical fiber sensing probe for detecting a carcinoembryonic antigen using a composite sensitive film of PAN nanofiber membrane and gold nanomembrane. Opt. Express.

[117] Xiao A., Huang Y., Zheng J., Chen P., Guan B.-O. (2019). An Optical Microfiber Biosensor for CEACAM5 Detection in Serum: Sensitization by a Nanosphere Interface. ACS Appl. Mater. Interfaces.

[118] Hu J., He P., Zhao F., Lin W., Xue C., Chen J., Yu Z., Ran Y., Meng Y., Hong X. (2024). Magnetic microspheres enhanced peanut structure cascaded lasso shaped fiber laser biosensor for cancer marker-CEACAM5 detection in serum. Talanta.

[119] Zhang Q., Zhao J., Han A., Zhang X., Yang M., Li H., Yu B., Zhang G., Zhou S. (2024). Ultrasensitive cancer cell sensing based on tapered optical fiber operating near the dispersion turning point. Sens. Actuators B Chem..

[120] Shaimerdenova M., Ayupova T., Ashikbayeva Z., Bekmurzayeva A., Blanc W., Tosi D. (2023). Reflector-Less Shallow-Tapered Optical Fiber Biosensors for Rapid Detection of Cancer Biomarkers. J. Light. Technol..

[121] Myrkhiyeva Z., Kantoreyeva K., Bekmurzayeva A., Gomez A.W., Ashikbayeva Z., Tilegen M., Pham T.T., Tosi D. (2024). Dynamic Measurement of a Cancer Biomarker: Towards In Situ Application of a Fiber-Optic Ball Resonator Biosensor in CD44 Protein Detection. Sensors.

[122] Yegizbay Z., Fatima M., Bekmurzayeva A., Ashikbayeva Z., Tosi D., Blanc W. (2025). Label-Free and Protein G-Enhanced Optical Fiber Biosensor for Detection of ALDH1A1 Cancer Biomarker. Fibers.

[123] Morales M.A., Halpern J.M. (2018). Guide to Selecting a Biorecognition Element for Biosensors. Bioconjugate Chem..

[124] Barbosa O., Ortiz C., Berenguer-Murcia Á., Torres R., Rodrigues R.C., Fernandez-Lafuente R. (2014). Glutaraldehyde in bio-catalysts design: A useful crosslinker and a versatile tool in enzyme immobilization. RSC Adv..

[125] Yang C. (2012). Enhanced physicochemical properties of collagen by using EDC/NHS-crosslinking. Bull. Mater. Sci..

[126] Sequeira-Antunes B., Ferreira H.A. (2023). Nucleic Acid Aptamer-Based Biosensors: A Review. Biomedicines.

[127] Zhao Y., Xin H., Wang C. (2024). Biomarker Multiplexing with Rational Design of Nucleic Acid Probe Complex. Anal. Sens..

[128] Liberti M.V., Locasale J.W. (2016). The Warburg Effect: How Does it Benefit Cancer Cells?. Trends Biochem. Sci..

[129] Pajak B., Siwiak E., Sołtyka M., Priebe A., Zieliński R., Fokt I., Ziemniak M., Jaśkiewicz A., Borowski R., Domoradzki T. (2019). 2-Deoxy-D-Glucose and its analogs: From diagnostic to therapeutic agents. Int. J. Mol. Sci..

[130] Cesewski E., Johnson B.N. (2020). Electrochemical biosensors for pathogen detection. Biosens. Bioelectron..

[131] Leibl N., Haupt K., Gonzato C., Duma L. (2021). Molecularly Imprinted Polymers for Chemical Sensing: A Tutorial Review. Chemosensors.

[132] Yang W., Ma Y., Sun H., Huang C., Shen X. (2022). Molecularly imprinted polymers based optical fiber sensors: A review. TrAC Trends Anal. Chem..

[133] Chen X., Noy A. (2021). Antifouling strategies for protecting bioelectronic devices. APL Mater..

[134] Gundagatti S., Srivastava S. (2023). An optimization of blocking agents for designing reliable electrochemical biosensors for ovarian cancer. Mater. Today Proc..

[135] Almeida L.C., Frade T., Correia R.D., Niu Y., Jin G., Correia J.P., Viana A.S. (2021). Electrosynthesis of polydopamine-ethanolamine films for the development of immunosensing interfaces. Sci. Rep..

[136] Chiang C.-Y., Chen C.-H., Wu C.-W. (2023). Fiber Optic Localized Surface Plasmon Resonance Sensor Based on Carboxymethylated Dextran Modified Gold Nanoparticles Surface for High Mobility Group Box 1 (HMGB1) Analysis. Biosensors.

[137] Ucci S., Spaziani S., Quero G., Vaiano P., Principe M., Micco A., Sandomenico A., Ruvo M., Consales M., Cusano A. (2022). Advanced Lab-on-Fiber Optrodes Assisted by Oriented Antibody Immobilization Strategy. Biosensors.

[138] Hasler R., Vísová I., Vrabcová M., Houska M., Spasovová M., Lísalová H., Dostálek J., Canva M.T., Giannetti A., Altug H., Moreau J. (2022). Fiber optic probe with antifouling polymer brush biointerface for bi-modal biosensing in complex liquid samples. Biophotonics in Point-of-Care II.

[139] Qian H., Huang Y., Duan X., Wei X., Fan Y., Gan D., Yue S., Cheng W., Chen T. (2019). Fiber optic surface plasmon resonance biosensor for detection of PDGF-BB in serum based on self-assembled aptamer and antifouling peptide monolayer. Biosens. Bioelectron..

[140] D’agata R., Bellassai N., Jungbluth V., Spoto G. (2021). Recent Advances in Antifouling Materials for Surface Plasmon Resonance Biosensing in Clinical Diagnostics and Food Safety. Polymers.

[141] Völlmecke K., Afroz R., Bierbach S., Brenker L.J., Frücht S., Glass A., Giebelhaus R., Hoppe A., Kanemaru K., Lazarek M. (2022). Hydrogel-Based Biosensors. Gels.

[142] Sitinjak N.A., Huang C.-W., Yang T.-Y., Chau L.-K., Wang C.-H. (2025). Synthesis of Carboxymethyl Dextran-Coated Gold Nanoparticles as Stable and Storable Optical Labels for Ultrasensitive Plasmonic Nanoparticle-Linked Sorbent Assay. Sensors.

[143] Mortazavi S., Makouei S., Abbasian K., Danishvar S. (2025). Emerging Trends in Optical Fiber Biosensing for Non-Invasive Biomedical Analysis. Photonics.

[144] Malakar T., Amina M.N.N., Nijhum Z.T., Lalin N.S. (2025). Optimized Single-Core PCF-Based SPR Biosensor for High-Performance Early-Stage Multi-Cancer Detection. arXiv.

[145] Chaudhary V.S., Kumar D., Kumar S. (2022). Au-TiO_2_ Coated Photonic Crystal Fiber Based SPR Refractometric Sensor for Detection of Cancerous Cells. IEEE Trans. Nanobiosci..

[146] Hu J., Song E., Liu Y., Yang Q., Sun J., Chen J., Meng Y., Jia Y., Yu Z., Ran Y. (2023). Fiber Laser-Based Lasso-Shaped Biosensor for High Precision Detection of Cancer Biomarker-CEACAM5 in Serum. Biosensors.

[147] Khani S., Hayati M. (2022). Optical biosensors using plasmonic and photonic crystal band-gap structures for the detection of basal cell cancer. Sci. Rep..

[148] Chaity A.C. (2023). Highly Sensitive Photonic Crystal Fiber Biosensor Based on Surface Plasmon Resonance for Six Distinct Types of Cancer Detection. Plasmonics.

[149] Dashtmian K., Reza M., Fallahi V., Seifouri M., Olyaee S. (2025). Gold Nanowire-Enhanced SPR-PCF Biosensor for High-Throughput Cancer Cell Detection in Near-Infrared. Plasmonics.

[150] Hossain B., Akib T.B.A., Abdulrazak L.F., Rana M. (2019). Numerical modeling of graphene-coated fiber optic surface plasmon resonance biosensor for BRCA1 and BRCA2 genetic breast cancer detection. Opt. Eng..

[151] Verma P., Kumar A., Jindal P. (2022). Machine Learning Approach for SPR based Photonic Crystal Fiber Sensor for Breast Cancer Cells Detection. Proceedings of the 2022 IEEE 7th Forum on Research and Technologies for Society and Industry Innovation (RTSI).

[152] Sobur T.R., Hasan M., Khatun M.R., Mia M., Islam W., Iqbal S., Hossain M.M. (2025). Machine learning-enhanced SPR-based optical biosensor for cancer cell detection. Opt. Laser Technol..

[153] Wekalao J., Li M., Zhang F., Zhang X., Liu W. (2025). Machine learning-enhanced Kretschmann configuration plasmonic biosensor for real-time brain tumor biomarker detection. Surf. Interfaces.

[154] Ashrafian M., Olyaee S., Seifouri M. (2025). Highly sensitive cancer detection using an open D-channel PCF-based SPR biosensor. Sci. Rep..

[155] Zhang Y., Miao T., Mu Q., Zhou L., Meng C., Xue J., Yao Y. (2022). A Novel High-Sensitivity Terahertz Microstructure Fiber Biosensor for Detecting Cancer Cells. Photonics.

[156] Lv J., Wang J., Yang L., Liu W., Fu H., Chu P.K., Liu C. (2024). Recent advances of optical fiber biosensors based on surface plasmon resonance: Sensing principles, structures, and prospects. Sens. Diagn..

[157] Bissen A., Yunussova N., Myrkhiyeva Z., Salken A., Tosi D., Bekmurzayeva A. (2024). Unpacking the packaged optical fiber bio-sensors: Understanding the obstacle for biomedical application. Front. Bioeng. Biotechnol..

[158] SDS Optic S.A. Starts Collaboration with Accrea Medical Robotics sp. Zoo and MedApp S.A. https://www.sdsoptic.pl/.

[159] Detection of Breast Cancer by Fluorescence Using an Optical Fiber Needle. https://www.sedi-ati.com/business_cases/detection-of-breast-cancer-by-fluorescence-using-an-optical-fiber-needle/.

[160] Zhu G., Zhang M., Lu L., Lou X., Dong M., Zhu L. (2019). Metal-organic framework/enzyme coated optical fibers as waveguide-based biosensors. Sens. Actuators B Chem..

[161] Karimian S., Ali M.M., McAfee M., Saleem W., Duraibabu D., Memon S.F., Lewis E. (2025). Challenges in Adapting Fibre Optic Sensors for Biomedical Applications. Biosensors.

